# Mechanobiological Adaptation to Hyperosmolarity Enhances Barrier Function in Human Vascular Microphysiological System

**DOI:** 10.1002/advs.202206384

**Published:** 2023-02-19

**Authors:** Joon Ho Kang, Minjeong Jang, Su Jin Seo, Andrew Choi, Daeeun Shin, Suyoung Seo, Soo Hyun Lee, Hong Nam Kim

**Affiliations:** ^1^ Brain Science Institute Korea Institute of Science and Technology Seoul 02792 Republic of Korea; ^2^ Department of Chemical Engineering Kwangwoon University Seoul 01897 Republic of Korea; ^3^ School of Mechanical Engineering Sungkyunkwan University Suwon 16419 Republic of Korea; ^4^ Program in Nano Science and Technology Graduate School of Convergence Science and Technology Seoul National University Seoul 08826 Republic of Korea; ^5^ Division of Bio‐Medical Science & Technology KIST School University of Science and Technology (UST) Seoul 02792 Republic of Korea; ^6^ School of Mechanical Engineering Yonsei University Seoul 03722 Republic of Korea; ^7^ Yonsei‐KIST Convergence Research Institute Yonsei University Seoul 03722 Republic of Korea

**Keywords:** 3D human vascular microphysiological system, hyperosmolarity, inflammation, mechanobiology, vascular barrier function, Yes‐associated protein (YAP)

## Abstract

In infectious disease such as sepsis and COVID‐19, blood vessel leakage treatment is critical to prevent fatal progression into multi‐organ failure and ultimately death, but the existing effective therapeutic modalities that improve vascular barrier function are limited. Here, this study reports that osmolarity modulation can significantly improve vascular barrier function, even in an inflammatory condition. 3D human vascular microphysiological systems and automated permeability quantification processes for high‐throughput analysis of vascular barrier function are utilized. Vascular barrier function is enhanced by >7‐folds with 24–48 h hyperosmotic exposure (time window of emergency care; >500 mOsm L^−1^) but is disrupted after hypo‐osmotic exposure (<200 mOsm L^−1^). By integrating genetic and protein level analysis, it is shown that hyperosmolarity upregulates vascular endothelial‐cadherin, cortical F‐actin, and cell–cell junction tension, indicating that hyperosmotic adaptation mechanically stabilizes the vascular barrier. Importantly, improved vascular barrier function following hyperosmotic exposure is maintained even after chronic exposure to proinflammatory cytokines and iso‐osmotic recovery via Yes‐associated protein signaling pathways. This study suggests that osmolarity modulation may be a unique therapeutic strategy to proactively prevent infectious disease progression into severe stages via vascular barrier function protection.

## Graphical abstract

This study reports that osmolarity modulation can significantly improve vascular barrier function, even in an inflammatory condition by using 3D human vascular microphysiological systems and automated permeability quantification processes.

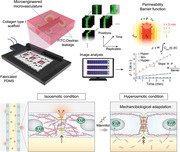

## 1 Introduction

Leaky blood vessels are major underlying causes of various cardiovascular diseases as the vascular barrier integrity is essential in regulating fluid homeostasis, macromolecular and cellular transport, and inflammation.^[^ ^1^ ^]^ For instance, in infectious diseases such as COVID‐19 and sepsis that can lead to death as short as 12–48 h,^[^ ^2^ ^]^ hyperproduction of inflammatory cytokines, termed hypercytokinemia or the “cytokine storm,” is often accompanied by severe blood vessel leakage.^[^ ^3^ ^]^ Impaired vascular barrier function may trigger hypotension, hypoperfusion, and uncontrolled leakage of inflammatory cytokines, which may cause fatal outcomes such as multi‐organ failure, and ultimately death.^[^ ^4^ ^]^ Hence, preventing blood vessel leakage has been recognized as the key to delaying the progression of such lethal conditions,^[^ ^5^ ^]^ but therapeutic strategies to protect or improve the blood vessel barrier function are currently limited.

Several studies have reported the barrier‐enhancing and protecting effects of hyperosmolar treatments. For instance, acute exposure to a hyperosmotic sucrose solution in excised murine lung capillaries reduce the hydraulic conductivity across capillaries.^[^ ^6^ ^]^ In addition, hypertonic saline or mannitol infusion is often implemented in the clinic (e.g., osmotherapy) to intervene with serious hemorrhage^[^ ^7^ ^]^ as well as traumatic brain injury via acute exposure,^[^ ^8^ ^]^ although their effectiveness has not been fully established.

Paradoxically, the infusion of hyperosmotic agents, such as mannitol, is perhaps the most widely used method to temporarily disrupt the blood vessel barrier and increase vascular permeability.^[^ ^9^ ^]^ Particularly, hyperosmolarity is frequently used to promote chemotherapeutic agent delivery across the blood–brain barrier (BBB) and to the target sites in the brain.^[^ ^10^ ^]^ The hypertonicity triggers endothelial cell shrinkage^[^ ^11^ ^]^ and consequently leads to physical cell‐to‐cell junction breakage, thereby “opening” the blood barrier.^[^ ^12^ ^]^ As such, mixed reports exist regarding the effect of hyperosmolarity on the blood vessel barrier function. Moreover, the effect of hypo‐osmolarity on vascular barrier function remains unknown.

However, studying the effect of osmolarity on vascular barrier function in vivo, presents two major challenges. i) Osmolarity in living organisms is highly homeostatic;^[^ ^13^ ^]^ plasma osmolarity, other than in renal system, is tightly maintained within a few percent,^[^ ^14^ ^]^ and as such it is especially challenging to control and maintain the desired osmolarity for extended periods of time. ii) Osmolarity affects the microenvironment surrounding the blood vessel (e.g., immune activation/suppression,^[^ ^15^ ^]^ blood cell size and stiffness,^[^ ^16^ ^]^ blood viscosity,^[^ ^17^ ^]^ and platelet aggregation^[^ ^18^ ^]^) which in turn may affect the vascular integrity, independent of the osmolarity.

Alternatively, ex vivo^[^ ^6^ ^]^ and in vitro approaches are relatively free from the above complications. In vitro approaches are especially appealing to study barrier properties, as i) the geometry and dimension of blood vessels can be precisely designed, ii) different types of endothelial cells can be used, iii) barrier functions (i.e., vascular permeability) can be precisely and rapidly quantified, and (iv) in‐depth biological analysis can be robustly performed. In addition to conventional transwell assays,^[^ ^19^ ^]^ a wide variety of 3D microvasculature engineering techniques have been developed.^[^ ^20^ ^]^ Implementing microfluidic channel fabrication^[^ ^21^ ^]^ and 3D printing techniques,^[^ ^22^ ^]^ numerous studies have mimicked in vivo vascular functions for human disease modeling. Recently, culturing monolayers of endothelial cells onto 3D hollow cylindrical spaces in a collagen scaffold was used to study diverse pathological and cardiovascular conditions.^[^ ^23^ ^]^ However, measurement throughput and feasibility are limited for large‐scale screening and modulation of complex vascular conditions.

In this study, we advance 3D microvasculature engineering to quantify microvessels barrier function with significantly improved throughput and feasibility. Integration of an automated image acquisition and analysis system allowed systematical investigation of the cause and consequences of osmolarity (150–600 mOsm L^−1^) induced changes in vascular permeability. We show that temporal osmolarity control around the engineered microvasculature may regulate the vascular barrier function, thereby providing a “time window” for the use of hyperosmotic agents for enhancing vascular barrier function. In addition, we validate that such a barrier‐enhancing strategy can be adapted to various inflammatory conditions and could potentially be used as a therapeutic method for vascular protection in hypercytokinemia. Finally, we investigate the underlying mechanism of osmolarity‐controlled vascular barrier protection by genetic and protein level analysis.

## 2 Results

### 2.1 High‐Throughput Human Microvasculature Engineering Platforms

**Figure 1:**
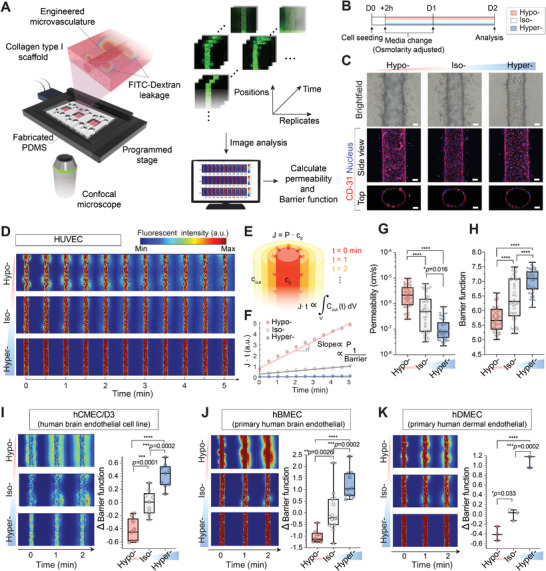
Continuous hyperosmolarity exposure enhances the vascular barrier function in human microvasculature‐on‐chips. A) Human microvessel engineering and barrier function assay platform. Endothelial cells are cultured onto a cylindrical collagen scaffold. The barrier functions of engineered microvessels are then quantified by time‐course imaging of fluorescein isothiocyanate (FITC)‐dextran leakage out from the vessel lumen. B) Experimental timeline for testing the effect of osmotic exposure on microvessel barrier function. Otherwise noted, all images and data represent microvessels 2 d after osmolarity adjustments (D2). C) Representative bright‐field images (top) and immunostaining of CD31 images (bottom; side and top view) of osmolarity (hypo‐, iso‐, or hyperosmolarity) adapted human umbilical vein endothelial cell (HUVEC) 3D engineered microvessels. Cell nuclei were counterstained with DAPI. For iso‐osmotic conditions, microvessels are cultured in regular endothelial culture media. Note that cylindrical lumen (hollow channel) is created inside the vessels. Scale bars, 50 µm. D) Representative fluorescent images of 4 kDa FITC‐dextran leakage from osmolarity‐adapted HUVEC engineered microvessels. Three images along the vessel's vertical positions were acquired per each microvessel. *t* = 0 min images were taken immediately after the lumen was filled with 4 kDa FITC dextran. E,F) Schematic and representative dextran flux graph as a function of time from osmolarity‐adapted HUVEC 3D engineered microvessels. Permeability (and 1/Barrier function; values > 0) is quantified from the slope of the *J*∙*t* versus *t* graph. G,H) Permeability and barrier function of osmolarity‐adapted HUVEC 3D engineered microvessels at D2 (*n* = 48, 50, and 47 engineered microvessels for hypo‐, iso‐, and hyperosmotic conditions, respectively). I–K) Representative fluorescent images of 4 kDa FITC‐dextran leakage (left) and barrier function (right) of osmolarity‐adapted hCMEC/D3, hBMEC, and hDMEC 3D engineered microvessels (hCMEC/D3: *n* = 9, 9, and 9; hBMEC: *n* = 9, 15, and 10; hDMEC: *n* = 3, 3, and 3, for hypo‐, iso‐ and hyper‐, respectively). Box and whisker plots in panel (G)–(K) represent median value (horizontal bars), 25–75 percentiles (box edges), and minimum to maximum values (whiskers). *P*‐values were obtained using one‐way ANOVA followed by Tukey's HSD post hoc test. n.s.: not significant, ****P < 0.0001.

We developed a 3D vascular engineering platform to study the effect of osmotic exposure on human endothelial vasculature. We used in vitro vascular microphysiological systems composed of polydimethylsiloxane (PDMS) chips with embedded chambers and microchannels, as described in previous literature.^[^ ^23a^ ^]^ Briefly, collagen was injected into the rectangular chamber with a dimension of 5 × 10 × 1.4 mm (*W* × *D* × *H*) partially occupied with pre‐inserted microneedles. After collagen gelation, the needles were removed and human endothelial cells were subsequently infused into the center hollow cylindrical channels, which were initially occupied by one of the needles (Experimental Section). Endothelial cells immediately adhered to the hollow channel walls upon infusion and formed endothelium in vitro (**Figure** **1**A, *left*). Cells were continuously supplied with the culture medium through the cylindrical lumen and two parallel microchannels on each side. The endothelia were cultured under static flow conditions and were only exposed to gravity‐driven perfusion during the time of initial cell seeding and at the time of regular media exchange.^[^ ^23c^ ^]^ The gravity‐driven perfusion led to a flow rate <5 nL s^−1^, consequently exposing vasculatures to a maximum shear stress of ≈40 dyne cm^−2^. The flow eventually ceased after <30 min, as the heights of the fluids in the two reservoirs were balanced. Following barrier maturation, vascular permeability was evaluated by imaging lumen‐infused 4 kDa fluorescein isothiocyanate (FITC)‐dextran leakage to the surrounding collagen scaffold. In this study, we expanded the 3D vascular engineering platform to be capable of i) culturing three independent microvessels per device, and ii) measuring three vertical positions per microvessel simultaneously, consequently increasing the throughput of the barrier function analysis by ≈10× (Figure 1A, *right*).

Roughly 2 h after cell infusion, we exchanged the media with hypo‐ (150 mOsm L^−1^; culture media + H_2_O), iso‐ (300 mOsm L^−1^; media), or hyper‐osmotic (600 mOsm L^−1^; media + mannitol) media (Experimental Section). Endothelium was then cultured for an additional 24–48 h in the osmolarity‐adjusted media, followed by automated barrier function analysis (Figure 1B). All microvasculature‐on‐chips adapted to hypo‐, iso‐, and hyperosmotic conditions displayed cylindrical morphology with a hollow lumen (Figure 1C, *bottom*). In addition, all microvessels were stained positive with CD31, indicating successful barrier maturation across all osmotic conditions (Figure 1C). We did not observe major changes in viability or proliferation patterns between the microvessels adapted to hypo‐, iso‐, and hyperosmotic conditions (Figure S1, Supporting Information).

### 2.2 Hyperosmolarity Exposure Improves Vascular Barrier Function

We questioned whether microvessels adapted to continuous hyper‐ or hypo‐osmolarity exposure displayed any changes in their barrier function, or more specifically, vascular permeability. To test this, we sequentially imaged the hypo‐, iso‐, and hyperosmolarity adapted human umbilical vein endothelial cell (HUVEC) microvasculature‐on‐chips immediately after the infusion of 4 kDa FITC‐dextran solution into the vessel lumen (Figure 1D). The osmolarity of the 4 kDa FITC‐dextran solution was matched with that of the culture medium to which the microvessels were adapted (Experimental Section). We assumed that the 4 kDa FITC‐dextran leakage follows mass transport dynamics across the semipermeable cylindrical membrane of infinite source (Figure 1E; Note S1, Supporting Information; and Experimental Section). Under this assumption, the total amount of barrier‐crossed 4 kDa FITC‐dextran at a given time point (*t*), which can be calculated by integrating total fluorescence intensity outside the cylindrical lumen (Figure 1F; $\int_{V}C_{out}\left(t\right)dV$), will be proportional to the flux (*J* = *Pc_0_*) across the barrier. Thus, the dextran permeability (P) could be quantified by the slope of the line in the *J* · *t* versus *t* graph (Figure 1F, and Figure S2, Supporting Information). Given the fast time scale of the 4 kDa dextran leakage (≈1 min), it is likely that the permeability of the microvessels is not of transcellular (i.e., uptake and release of the dyes by the cells)^[^ ^24^ ^]^ origin, but rather of paracellular (i.e., between the cells) origin.^[^ ^25^ ^]^ The molecular weight of 4 kDa was chosen over higher molecular weight dextrans to increase the sensitivity of our vascular permeability analysis, as some hyperosmolarity‐adapted microvessels displayed 4 kDa dextran permeability close to the lower limit of detection.

The vascular permeability inversely correlated with the osmolarity of the media to which the microvessels were adapted; Hyper‐ displayed a 7.44 ± 1.84‐fold decrease, whereas hypo‐ displayed a 2.69 ± 0.61‐fold increase in the vascular permeability when compared to iso‐osmotic controls (Figure 1G). The collagen fiber structures (Figure S3A, Supporting Information) and the permeability of FITC‐Dextran across the hollow channels without any endothelial cell (Figure S3B–D, Supporting Information) were not affected by osmotic perturbations. Importantly, similar permeability changes were observed in the osmolarity‐adapted HUVEC monolayers cultured on collagen surface‐coated Transwell plates (2D assays; Figure S4A,B, Supporting Information), as well as those cultured on thick, gelated collagen beds (2.5D assays; Figure S4C, Supporting Information).

Consistent with previous works,^[^ ^23c^ ^]^ the vascular permeability followed an approximately log‐normal distribution (Figure S2G, Supporting Information). We defined a new index, barrier function, as a negative logarithm of the permeability so that the newly defined quantity positively correlates with the vascular barrier properties:

(1) $$\mathrm{Barrier}\;\mathrm{function}=-\log_{10}\;\left[\mathrm{Permeability}\right]$$

Thus, a tenfold decrease and increase in the vascular permeability will correspond to +1 and −1 change in barrier functions, respectively. The barrier function of hypo‐ and hyperosmolarity‐adapted microvessels displayed −0.60 ± 0.12 and 0.66 ± 0.12 change, respectively, relative to the barrier function of the iso‐osmotic microvessels (Figure 1H). Hyperosmotic sorbitol exposure induced similar improvements in the microvasculature barrier function (Figure S5A,B, Supporting Information). In addition, the positive correlation between the osmolarity and barrier function of microvasculature‐on‐chip was observed across a wide range of osmolarity (150–600 mOsm L^−1^; Figure S6A,B, Supporting Information), and across multiple days after the osmotic modulation (days 1–4; Figure S7, Supporting Information). However, hyperosmotic shock following barrier maturation, whether acute (30 min) or long‐term (24 h), did not improve the barrier function (Figure S8, Supporting Information), suggesting that hyperosmolarity‐induced barrier function improvement require early exposure.

Notably, the osmolarity‐dependent change in barrier function was universally observed across microvessels engineered from multiple human endothelial cell types. Human cerebral microvascular endothelial cell line (hCMEC/D3; Figure 1I), human brain microvascular endothelial cell (hBMEC; Figure 1J), and human dermal microvascular endothelial cell (hDMEC; Figure 1K), derived microvessels displayed −0.43 ± 0.06, −1.02 ± 0.09, and −0.40 ± 0.08 impairment in barrier functions after hypo‐, respectively, and 0.41 ± 0.06, 1.23 ± 0.19, and 1.14 ± 0.09 improvement in barrier functions after hyperosmolarity adaption, respectively.

### 2.3 Osmolarity Induces Distinct Cell–Cell Junction Gene Expression

**Figure 2:**
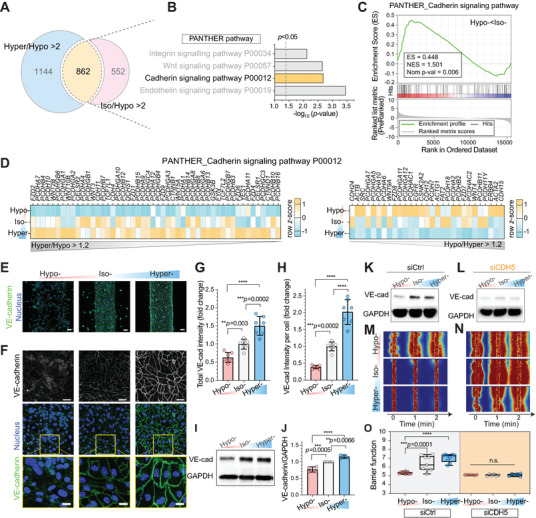
Adherent junction drives osmolarity‐driven barrier function change. A) Number of genes that are upregulated by more than twofolds in hyper‐ (Hyper/Hypo > 2) and iso‐ (Iso/Hypo > 2) compared to hypo‐osmotic conditions. B) Gene ontology (GO) analysis of the 862 intersected genes. Significantly enriched gene sets were selected from the PANTHER pathway. The dashed vertical lines indicate significance at *p* < 0.05. C) Gene set enrichment analysis (GSEA) results showing significant enrichment of the gene sets, “Cadherin signaling pathway” in Reactome from the Molecular Signatures Database (MSigDB) in iso‐ compared to hypo‐osmotic conditions. Red and blue shading indicate high and low log_2_‐ranked values comparing iso‐ to hypo‐osmotic conditions, respectively. ES: enrichment score, NES: normalized enrichment score, and Nom *p*‐value: nominal *p*‐value. D) Heatmap visualization of gene expression profiles of PANTHER_Cadherin signaling pathway (P00012). Genes over 1.2‐fold up‐ (Hyper/Hypo > 1.2) and downregulated (Hypo/Hyper > 1.2) in hyper‐ compared to hypo‐osmotic conditions are displayed based on the *z*‐score. E,F) Representative immunostaining of VE‐cadherin in HUVEC 3D engineered microvessels and 2.5D monolayer (see the Experimental Section) 2 d after corresponding osmolarity adjustment (hypo‐, iso‐, and hyperosmolarity at D2; see Figure 1B for detailed timelines). Cell nuclei were counterstained with DAPI. Scale bars, 50 µm. Inset: zoom‐in view of cell junctions. Scale bars, 10 µm. G,H) Total VE‐cadherin intensity and intensity per cell from immunostained images relative to iso‐osmotic condition. Mean ± S.D. *n* = 7 images from three biological replicates. I) Representative western blot displaying VE‐cadherin levels of 2.5D HUVEC monolayers at D2. GAPDH was used as a loading control. J) Western blot‐based quantification of VE‐cadherin levels relative to iso‐osmolarity conditions. VE‐cadherin levels were normalized by GAPDH level. Mean ± S.D. *N* = 4 independent experiments. K,L) Western blot displaying VE‐cadherin levels of osmolarity‐adapted control siRNA (siCtrl) and siCDH5‐treated HUVEC cells. GAPDH was used as the loading control. M,N) Representative fluorescent images of 4 kDa FITC‐dextran leakage from siCtrl and siCDH5‐treated HUVEC 3D engineered microvessels after osmolarity adaptation. Cells in culture were treated with siCtrl or siCDH5 for 2 d before cell seeding. See Figure S13 (Supporting Information) for detailed timelines. *t* = 0 min images were taken immediately after the lumen was filled with FITC‐dextran solutions. O) Barrier function of siCtrl (left) and siCDH5 (right) treated HUVEC 3D engineered microvessels after osmolarity adaptation (siCtrl: *n* = 9, siCDH5: *n* = 6 microvessels for each osmolarity condition). Box and whisker plots represent median value (horizontal bars), 25–75 percentiles (box edges), and minimum to maximum values (whiskers). For panels (G), (H), (J), and (O), *P*‐values were obtained using one‐way ANOVA followed by Tukey's HSD post hoc test. n.s: not significant, ****P < 0.0001.

Next, we examined whether hypo‐ or hyperosmolarity‐adapted microvessels displayed any change in gene expression related to vessel integrity and cell–cell junction regulation. We assessed the global transcriptomic gene expression profile of HUVEC under hypo‐, iso‐, and hyperosmotic conditions using RNA‐sequencing (RNA‐seq). Assessment revealed that 862 intersected genes of over twofold upregulated in hyper‐ and iso‐ compared to the hypo‐osmotic conditions (**Figure** **2**A) are significantly associated with the “Cadherin signaling pathway (P00012)” gene set in the PANTHER pathway (Figure 2B), which was also positively enriched in iso‐ compared to hypo‐osmotic conditions (Figure 2C). There were more increased upregulated genes compared to downregulated genes among the P00012 gene sets in hyper‐ compared to hypo‐osmotic conditions (Figure 2D). Moreover, “Cell‐Cell junction (GO:0005911)” in the gene ontology (GO)_cellular components (GOCC) were positively enriched in hyper‐ compared to hypo‐osmotic conditions (Figure S9A, Supporting Information). Tight junction‐related gene sets in GOCC were significantly associated with hyper‐ compared to hypo‐osmotic conditions (Figure S9B,C, Supporting Information). We observed consistent changes in tight junction proteins, zonula occludens‐1 (ZO‐1; Figure S9D, Supporting Information), and its expression levels (Figure S9E, Supporting Information). For all hypo‐, iso‐, and hyperosmolarity adapted microvessels, the adherens junctional protein and vascular endothelial cadherin (VE‐cadherin, *CDH5*) expression were significantly higher than ZO‐1 (*TJP1*), both in proteins and their encoding mRNAs (Figure S9E,F, Supporting Information). Subsequently, we focused on the VE‐cadherin expression level and localization.

### 2.4 VE‐Cadherin Upregulation Following Hyperosmotic Exposure

The 3D microvasculatures‐on‐chip from various human endothelial origins displayed consistent VE‐cadherin expression and localization after hypo‐ and hyperosmolarity adaptation (Figure 2E, and Figure S10, Supporting Information). Qualitatively, all hypo‐osmolarity‐adapted microvessels showed weaker and disordered VE‐cadherin expression. In contrast, hyperosmolarity‐adapted microvessels displayed stronger and uniform VE‐cadherin structure near cell–cell boundaries (Figure 2E, and Figure S10, Supporting Information). We then aimed to quantify microstructure changes in the cell–cell junction following osmolarity adaptation. However, 3D microvasculature‐on‐chip imaging, by its inherent design, required exceptionally long and wide ranges of optical working distance, and consequently, high‐resolution imaging and quantification of the cell–cell junction structures were not practically feasible. To this end, we cultured endothelial monolayers on thick, gelated collagen beds (i.e., 2.5D, Figure S4A, Supporting Information). We used the same collagen composition and protocol used for 3D microvessel engineering and confirmed that cells in 2.5D and 3D display similar tendencies in their barrier function and VE‐cadherin expression profile after osmolarity adaptation (Figures 1G and 2E,F, and Figure S4C, Supporting Information).

Both the total VE‐cadherin intensity levels (i.e., total intensity in image area; Figure 2G) and VE‐cadherin intensity per cell (total intensity/number of cells; Figure 2H) in 2.5D HUVEC cells significantly correlated with the osmolarity to which they were adapted (Figure 2G,H, and Figure S6C, Supporting Information). The total VE‐cadherin intensity decreased by 37 ± 5% and increased by 50 ± 10% for hypo‐ and hyper‐, respectively, compared to the iso‐osmotic controls. The VE‐cadherin intensity per cell decreased by 61 ± 2% and increased by 103 ± 14% for hypo‐ and hyper‐, respectively, compared to the iso‐osmotic controls. Hyperosmotic sorbitol and NaCl yielded similar phenotypes (Figure S5C,D, Supporting Information). The VE‐cadherin expression level assayed by western blot (Figure 2I,J, and Figure S6D, Supporting Information) was consistent with the immunofluorescence quantification. Notably, the microstructure of the VE‐cadherin adherens junction was also drastically affected. Hypo‐ and iso‐osmolarity‐adapted monolayers often displayed holes and punctured VE‐cadherins (Figure S11, Supporting Information). However, the number of holes and nest‐like junctional phenotypes were dramatically reduced in hyperosmotic conditions, consequently leading to tighter and narrower cell–cell junctions (Figure S11E, Supporting Information). In addition, cell area and perimeter increased in hyper‐, but decreased in hypo‐osmolarity‐adapted 2.5D monolayers. The circularity, on the other hand, increased in hypo‐osmotic conditions (Figure S12A–F, Supporting Information). Similar changes in cell size were observed in 3D chip conditions (Figure S12G,H, Supporting Information).

These findings led us to wonder whether VE‐cadherin is necessary for osmolarity‐dependent changes in vascular barrier function. To test this, *CDH5* (VE‐cadherin)‐depleted HUVECs were constructed with small interfering RNAs (siCDH5). HUVECs were incubated with Control siRNA (siCtrl) or siCDH5 for 2 d prior to the cell seeding on‐chip (Figure S13, Supporting Information, and Experimental Section). siCDH5‐treated HUVECs displayed notable downregulation in VE‐cadherin levels even after continuous hyperosmolarity exposure and barrier maturation (Figure 2K,L, and Figure S13C–E, Supporting Information). The VE‐cadherin‐depleted (siCDH5) microvessels displayed significantly lower barrier function (i.e., increased vascular permeability; Figure 2N) in all osmotic conditions, compared to the control (siCtrl) microvessels (Figure 2M). Importantly, the barrier function improvements following hyperosmolarity adaptation previously observed in multiple endothelial cell type‐derived microvessels (Figure 1H–K) were completely abolished in siCDH5 microvessels (Figure 2O), implying that VE‐cadherin is required not only for maintaining but also for osmolarity‐dependent modulation in vascular barrier integrity.

### 2.5 Microvessels Mechanically Adapt during Hyperosmotic Exposure

**Figure 3:**
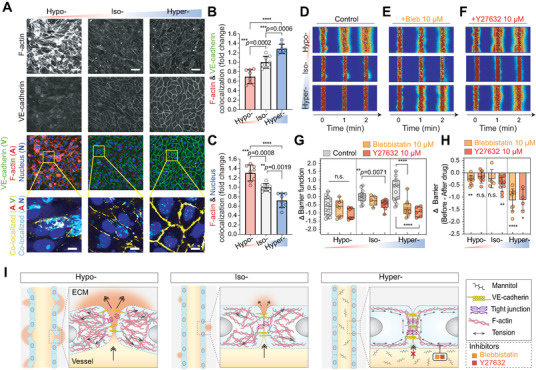
Cell–cell junction localization of F‐actin and actomyosin‐dependent barrier function imply mechanobiological adaptation of microvessels during osmolarity exposure. A) Representative immunostaining of F‐actin and VE‐Cadherin in HUVEC 2.5D monolayer 2 d after corresponding osmotic adjustment (hypo‐, iso‐, or hyperosmotic condition at D2; see Figure 1B for detailed timelines). Scale bars, 50 µm. Cell nuclei were counterstained with DAPI. Inset: zoom‐in view of F‐actin and VE‐cadherin (yellow) and actin and nucleus (cyan) colocalized pixels. Cell nuclei were counterstained with DAPI. Scale bars, 10 µm. B,C) Fraction of F‐Actin & VE‐cadherin and F‐Actin & Nucleus colocalized pixels from the immunostained images. Mean ± S.D. *n* = 8 images from five biological replicates. See Figure S15 (Supporting Information) for detailed processing steps. D–F) Representative fluorescent images of 4 kDa FITC‐dextran leakage from osmolarity‐adapted HUVEC 3D engineered microvessels without treatment, 30 min after 10 × 10^−6^ m Blebbistatin, and 10 × 10^−6^ m Y‐27632 treatment. *t* = 0 min images were taken immediately after the lumen was filled with FITC‐dextran solutions. G,H) Barrier function changes, relative to iso‐osmotic controls or before drug treatments, in osmolarity‐adapted HUVEC 3D engineered microvessels 30 min after 10 × 10^−6^ m Blebbistatin and 10 × 10^−6^ m Y‐27632 treatment (Control: *n* = 16, 14, and 13 microvessels for hypo‐, iso‐, and hyper‐, respectively; Blebbistatin: *n* = 9, 5, and 9 microvessels for hypo‐, iso‐, and hyper‐, respectively; Y‐27632: *n* = 7, 9, and 4 microvessels for hypo‐, iso‐, and hyper‐osmotic conditions, respectively). In panel (G), box and whisker plots represent median value (horizontal bars), 25–75 percentiles (box edges), and minimum to maximum values (whiskers). In panel (H), data represent Mean ± S.D. I) Proposed mechanism of osmolarity‐driven actin cytoskeletal change and its consequent effect on the vascular barrier function. For panels (B), (C), and (G), *P*‐values were obtained using one‐way ANOVA followed by Tukey's HSD post hoc test. In panel (H), *P*‐values obtained by two‐tailed, one‐sample *t*‐test compared to 0 (*P*‐values from left to right: 0.0013, 0.086, 0.22, 0.0012, <0.0001, 0.013). n.s: not significant, *P < 0.05, **P < 0.01, ***P < 0.001, ****P < 0.0001.

Since adherens junctions are interconnected to cell cytoskeleton,^[^ ^26^ ^]^ we questioned whether mechanical structures near cell–cell junction also rearrange during the osmolarity exposure. We specifically focused on cortical F‐actin localization, which are central mechanical backbones responsible for maintaining vascular barrier integrity.^[^ ^27^ ^]^ The RNA‐seq‐based analysis revealed that 903 intersected genes of 1.5‐fold upregulated in hypo‐ compared to hyper‐ and iso‐osmotic conditions are significantly associated with actin cytoskeleton‐related gene sets (Figure S14A–C, Supporting Information). Consistently, the total amount of F‐actins assayed both in immunofluorescence and western blots, inversely correlated with the osmolarity to which endothelial cells were adapted (**Figure** **3**A, and Figures S13C, S14D,E, and S15A, Supporting Information). In contrast, the F‐actin fraction localized in the cell–cell junction was proportional to the exposed osmolarity (Figure 3B; Figure S15, Supporting Information; and Experimental Section). The fraction of F‐actins localized with VE‐cadherins increased by 28 ± 4% for hyper‐ but decreased by 31 ± 5% for hypo‐osmolarity‐adapted microvessels. In contrast, nuclear localization of the F‐actins, analyzed by F‐actin and 4,6‐diamidino‐2‐phenylindole (DAPI) colocalization (Figure S15, Supporting Information), increased by 31 ± 6% for hypo‐ and decreased by 28 ± 5% for the hyper‐osmolarity‐adapted microvessels (Figure 3C). Therefore, F‐actins localize to the cell–cell junction rather than to the cell body during hyperosmolarity adaptation. Consistent with the previous works,^[^ ^6^ ^]^ depolymerizing F‐actins disrupted barrier integrity, even after osmolarity modulation (Figure S16, Supporting Information).

The cell–cell junction tension stabilizes the junction by reinforcing the bond between the cadherin–catenin complex and cortical actin.^[^ ^28^ ^]^ Hence, we wondered whether the junctional tension, at least partly, is responsible for the structural and functional changes in microvessel barriers during osmotic adaptation. To test this, we inhibited myosin II directly, and indirectly through rho‐associated protein kinase (ROCK) pathways. Surprisingly, hyperosmolarity‐induced improvements in the barrier function were completely abolished after acute treatment (30 min) with myosin II inhibitor Blebbistatin or ROCK inhibitor Y‐27632 (Figure 3D,F, and Figure S14F, Supporting Information). Hyperosmolarity‐adapted microvessels after Blebbistatin and Y‐27632 treatment decreased barrier function by 1.12 ± 0.15 and 1.10 ± 0.21, respectively, indicating more than tenfold increase in vascular permeability. Hypo‐ and iso‐osmolarity adapted microvessels, on the other hand, did not display major changes in the barrier function after the drug treatment (Figure 3G,H). Similar behavior was observed in 2.5D transwell cultured HUVEC monolayers acutely treated with Blebbistatin, but not with Y‐27632 (Figure S14G, Supporting Information). We suspect that the dramatically different structures and intensity levels of phosphorylated myosin light chain and actin localization,^[^ ^29^ ^]^ depending on endothelial barrier culture conditions (i.e., 2D, 2.5D, and 3D), impact the functional changes in the barrier (Figure S14H,I, Supporting Information). However, F‐actin localization to the cell–cell junction regions was not affected by drug treatments (Figure S17, Supporting Information), suggesting that actin polymerization and/or recruitments at the cell–cell junction occur gradually, beyond the timescale of acute inhibition (≈30 min). However, cell‐body accumulation of F‐actin stress fibers (indicated by the fraction of F‐actin co‐localized with the nucleus) was substantially reduced after both Y‐27632 and Blebbistatin treatment (Figure S17, Supporting Information). Therefore, consistent with previous reports,^[^ ^30^ ^]^ the stress fibers across the cell body can be acutely downregulated by myosin II inhibition within an hour. Altogether, our results imply that F‐actin colocalization and myosin‐dependent junctional tension are responsible for the hyperosmolarity‐induced improvements in the vascular barrier function (Figure 3I).

### 2.6 Hyperosmolar Protection against Vascular Inflammation

**Figure 4:**
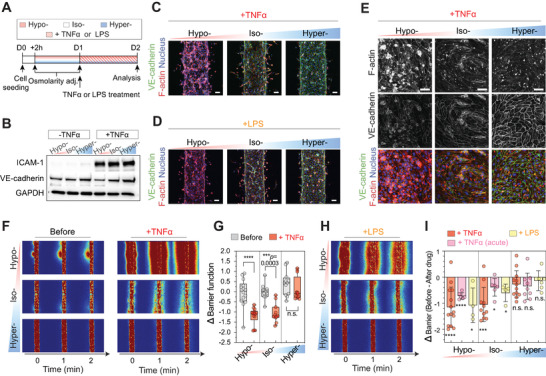
Hyperosmolarity‐adapted microvessels display significantly improved barrier protection under acute and chronic inflammation. A) Experimental timeline for testing the barrier protective effect of osmolarity‐adapted HUVEC 3D engineered microvessels following tumor necrosis factor alpha (TNF*α*) or lipopolysaccharides (LPS) induced vascular inflammations. Note that osmolarity was persistently maintained during inflammation. B) Western blot displaying ICAM‐1 (an inflammatory marker), and VE‐cadherin levels of osmolarity‐adapted (hypo‐, iso‐, or hyperosmotic) 2.5D HUVEC monolayers 24 h after 0 or 5 ng mL^−1^ TNF*α* treatment. GAPDH was used as a loading control. C–E) Representative immunostaining of VE‐cadherin and F‐actin in osmolarity‐adapted HUVEC 3D engineered microvessels 24 h after 5 ng mL^−1^ TNF*α*, 24 h after 100 ng mL^−1^ LPS treatment, and HUVEC 2.5D monolayer 24 h after 5 ng mL^−1^ TNF*α* treatment. Cell nuclei were counterstained with DAPI. Scale bars, 50 µm. F) Representative fluorescent images of 4 kDa FITC‐dextran leakage from osmolarity adjusted HUVEC 3D engineered microvessels before (left) and 24 h after 5 ng mL^−1^ TNF*α* (right) treatment. *t* = 0 min images were taken immediately after the lumen was filled with 4 kDa FITC dextran. G) Barrier function before and 24 h after 5 ng mL^−1^ TNF*α* treated microvessels with corresponding osmolarity adjustment. Data reflect change relative to iso‐osmotic conditions, before TNF*α* treatment. *n* = 14, 11, and 12 microvessels for hypo‐, iso‐, and hyperosmolarity, respectively. Box and whisker plots in panel (G) represent median value (horizontal bars), 25–75 percentiles (box edges), and minimum to maximum values (whiskers). *P*‐values were obtained using one‐way ANOVA followed by Tukey's HSD post hoc test. H) Representative fluorescent images of 4 kDa FITC‐dextran leakage from osmolarity‐adapted HUVEC 3D engineered microvessels 24 h after 100 ng mL^−1^ LPS treatment. *t* = 0 min images were taken immediately after the lumen was filled with 4 kDa FITC dextran. I) Barrier function changes of osmolarity‐adapted HUVEC 3D engineered microvessels 24 h after 5 ng mL^−1^ TNF*α* (*n* = 14, 11, and 12 microvessels for hypo‐, iso‐, and hyperosmotic conditions, respectively), 24 h after 100 ng mL^−1^ LPS (*n* = 4, 4, and 6 microvessels for hypo‐, iso‐, and hyperosmotic conditions, respectively), and 4 h after 100 ng mL^−1^ TNF*α* (acute; *n* = 6, 6, and 9 microvessels for hypo‐, iso‐, and hyperosmotic conditions, respectively). Data represent mean ± S.D. *P*‐values obtained by two‐tailed, one‐sample *t*‐test compared to 0 (*P*‐values from left to right: <0.0001, <0.0001, 0.0471, 0.0003, 0.037, 0.036, 0.092, 0.073, 0.42). For panels (G) and (I), n.s: not significant, *P < 0.05, **P < 0.01, ***P < 0.001, ****P < 0.0001.

In patients with severe inflammatory complications, such as COVID‐19 and sepsis, elevated proinflammatory cytokine levels, especially tumor necrosis factor‐*α* (TNF*α*),^[^ ^31^ ^]^ and lipopolysaccharides (LPS),^[^ ^32^ ^]^ are often accompanied by vascular hyperpermeability, which can lead to lethal septic shock.^[^ ^4^ ^]^ Recent research reports that proinflammatory mediators and cytokines, such as tumor necrosis factor‐*α* (TNF*α*), thrombin, and LPS, at least partially, mechanically disrupt endothelial cell–cell junctions.^[^ ^33^ ^]^ We wondered whether hyperosmolarity adaptation, which improves the barrier function through mechanical rearrangements and tension at the cell‐cell junction, in turn, can protect microvessels during chronic inflammation. To study this, we treated osmolarity‐adapted microvessels at D1 with TNF*α* or LPS (**Figure** **4**A, and Figure S18, Supporting Information) for 24 h to mimic the chronic exposure to pro‐inflammatory cytokines and mediators. The osmolarity was maintained during the TNF*α* or LPS treatment (Figure 4A). Microvessels adapted to all osmotic conditions were intercellular Adhesion Molecule 1 (ICAM‐1)‐positive after 24 h of TNF*α* treatment (Figure 4B). Hypo‐ and iso‐osmolarity adapted microvessels showed dramatic changes in their cell morphology as well as in cell–cell junction after the chronic TNF*α* (Figure 4C, and Figure S18C, Supporting Information) or LPS (Figure 4D) treatment. Following treatments, cells became notably narrow and elongated (Figure 4E, and Figure S18C, Supporting Information), consistent with a previous report.^[^ ^34^ ^]^ The VE‐cadherins in cell–cell junctions were generally less prominent and structured. The total F‐actin expression was significantly elevated and highly localized to the cell body, especially in hypo‐osmolarity‐adapted microvessels (Figure S17C, Supporting Information). Strikingly, cell morphology and VE‐cadherin expression patterns in hyperosmolarity‐adapted microvessels were not affected after the chronic TNF*α* or LPS exposure. Consistently, GO analysis revealed that the “Cadherin signaling pathway (P00012)” gene set in PANTHER pathway, is significantly related to highly expressed and maintained genes in hyper‐ compared hypo‐ and iso‐osmolarity‐adapted microvessels after the TNF*α* treatment (Figure S19A, Supporting Information). In addition, F‐actins remained highly localized to the cell‐cell junctions (Figure 4E, and Figure S17C, Supporting Information) similar to the untreated case (Figure 3A), suggesting that hyperosmolarity‐adapted microvessel may mechanically resist pro‐inflammatory cytokine or mediator‐driven vascular remodeling.

Next, we then investigated if microvessel hyperosmotic adaptation can prevent vascular leakage during chronic inflammation. Following TNF*α* 5 ng mL^−1^ 24 h treatment, hypervascular barriers remained tight, in contrast to hypo‐ or iso‐osmolarity adapted microvessels which showed significant leakage of the 4 kDa dextran FITC from the lumen (Figure 4F, and Figure S5E, Supporting Information). In fact, the barrier function of hyperosmolarity‐adapted microvessels was unchanged after chronic TNF*α* exposure (ΔBarrier = −0.28 ± 0.15; Figure 4G, and Figure S5F,G, Supporting Information). The barrier function of hypo‐ or iso‐osmolarity‐adapted microvessels decreased by 1.14 ± 0.20 and 1.05 ± 0.20, respectively, indicating that vessel leakage increased by ≈10‐fold for both conditions (Figure 4G). Similar barrier protective behavior was observed in hyperosmolarity‐adapted microvessels exposed to chronic LPS (1 µg mL^−1^ for 24 h; ΔBarrier = −0.14 ± 0.16; Figure 4H,I) and acute TNF*α* shock (100 ng mL^−1^ for 4 h; ΔBarrier = −0.35 ± 0.17; Figure 4I). Both hypo‐ and iso‐osmolarity‐adapted microvessels showed significantly elevated vascular leakage: ΔBarrier = −1.08 ± 0.33, −0.72 ± 0.05, −0.61 ± 0.17, and −0.39 ± 0.14 for hypo‐LPS, hypo‐acute TNF*α*, iso‐LPS, and iso‐acute TNF*α* microvessels, respectively. Therefore, our results suggest that hyperosmolarity adaptation act as a unique barrier protector against both chronic and acute vascular inflammation.

### 2.7 YAP‐Mediated Mechano‐Memory in Hyperosmolarity‐Adapted Microvessels

**Figure 5:**
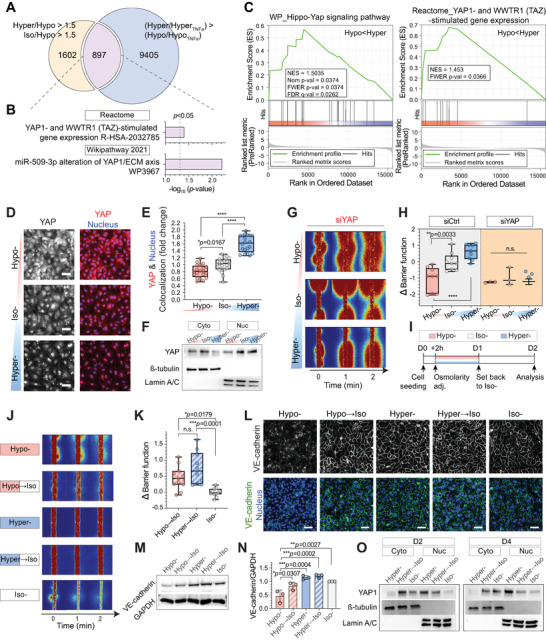
Hyperosmolarity‐induced Yes‐associated protein (YAP) nuclear localization and enhanced barrier integrity are sustained after iso‐osmotic recovery. A) Number of genes that are upregulated by more than 1.5‐folds in hyper‐ and iso‐ compared to hypo‐ (Hyper/Hypo > 1.5 and Iso/Hypo > 1.5) and that are highly maintained after TNF*α* treatment in hyper‐, but not in hypo‐osmotic conditions (Hyper/Hyper_TNF*α*_ > Hypo/Hypo_TNF*α*_). B) Gene ontology (GO) analysis for the 897 intersected genes from “Reactome” and “Wikipathway 2021.” The dashed vertical lines indicate significance at *p* < 0.05. C) Gene set enrichment analysis (GSEA) results showing significant enrichment of the gene sets, “Hippo‐Yap signaling pathway” in WikiPathways (WP) and “YAP1‐ and WWTR1 (TAZ)‐stimulated gene expression” in Reactome from the Molecular Signatures Database (MSigDB) in hyper‐ compared to hypo‐osmotic conditions. Red and blue shading indicate high and low log_2_‐ranked values comparing Hyper/Hypo. NES; normalized enrichment score, Nom *p*‐value; nominal *p*‐value, FWER; familywise‐error rate, FDR; false discovery rate. D) Representative immunostaining of YAP in HUVEC 2.5D monolayers 1 d after corresponding osmotic adjustment (hypo‐, iso‐, or hyperosmotic condition at D1; see Figure 1B for detailed timelines). Cell nuclei were counterstained with DAPI. Scale bars: 50 µm. E) Quantification of YAP and DAPI colocalization. *n* = 20 images from two independent experiments. F) Expression of cytoplasmic and nucleus YAP proteins in HUVEC 2.5D monolayers after osmolarity adaptation. ß‐tubulin and Lamin A/C were used as a loading control for cytoplasmic and nuclear proteins, respectively. G) Representative fluorescent images of 4 kDa FITC‐dextran leakage from osmolarity‐adapted HUVEC 3D engineered microvessels after siYAP treatment. Cells in culture were treated with siYAP 2 d before cell seeding. See Figure S13 (Supporting Information) for detailed timelines. H) Barrier function changes, relative to siCtrl iso‐osmotic conditions, in osmolarity‐adapted siCtrl (left; same as Figure 2G–K) and siCDH5 (right) treated HUVEC 3D engineered microvessels after osmolarity adaptation. I) Experimental timeline for testing the barrier function change of osmolarity‐adapted HUVEC 3D engineered microvessels following iso‐osmotic recovery (i.e., Hypo → Iso, Hyper → Iso). J) Representative fluorescent images of 4 kDa FITC‐dextran leakage from osmolarity‐adapted and iso‐osmotic recovered HUVEC 3D engineered microvessels. *t* = 0 min images were taken immediately after the lumen was filled with 4 kDa FITC‐dextran. K) Barrier function changes of osmolarity‐adapted HUVEC 3D engineered microvessels following iso‐osmotic recovery (i.e., Hypo → Iso, Hyper → Iso). Data reflect change relative to iso‐osmotic conditions at D2 (Iso‐). L) Representative immunostaining of VE‐cadherin in osmolarity‐adapted and iso‐osmotic recovered HUVEC 2.5D monolayers. Cell nuclei were counterstained with DAPI. Scale bars: 50 µm. M,N) Western blot images of total VE‐cadherin and quantifications of VE‐cadherin compared to GAPDH in osmolarity‐adapted and iso‐osmotic recovered HUVEC 2.5D monolayers. GAPDH was used as a loading control. Data represent mean ± S.D. *n* = 3 biological replicates. O) Expression of cytoplasmic and nuclear YAP in osmolarity‐adapted and iso‐osmotic recovered HUVEC 2.5D monolayers at D2 (left) and D4 (right). Note that nuclear YAP increase observed in hyper → iso samples at D2 finally recovers at D4. ß‐tubulin and Lamin A/C were used as a loading control for cytoplasmic and nuclear proteins, respectively. For panels (E), (H), and (K) box and whisker plots represent median value (horizontal bars), 25–75 percentiles (box edges), and minimum to maximum values (whiskers). For panels (E), (H), (K), and (N) *P*‐values were obtained using one‐way ANOVA followed by Tukey's HSD post hoc test. n.s: not significant, ****P < 0.0001.

To further unravel the upstream pathways responsible for improved barrier function and protection against inflammatory vessel leakage after hyperosmotic adaptation, we identified and classified highly expressed and maintained gene sets in hyper‐ compared to hypo‐ and iso‐osmolarity adapted microvessels following TNF*α* treatments (**Figure** **5**A, and Figure S19, Supporting Information). We sorted 897 genes by intersecting over 1.5‐fold upregulated genes in hyper‐ or iso‐ compared to hypo‐ (i.e., Hyper/Hypo and Iso/Hypo >1.5) and genes that are highly maintained after TNF*α* treatment in hyper‐, but not in hypo‐osmotic conditions (i.e., Hyper/Hyper_TNF*α*_ > Hypo/Hypo_TNF*α*_; Experimental Section). The intersected genes were also significantly associated with the YAP‐related gene sets in Reactome, Wikipathway, and CORUM (Figure 5B, and Figure S19C–E,G, Supporting Information). Additionally, the well‐known YAP‐target gene, *CTCF*, was significantly associated with the highly expressed and maintained genes in hyper‐ compared to the other osmotic conditions (Figure S19F–H, Supporting Information). Furthermore, we found that YAP‐related gene sets, including the “Hippo‐Yap signaling pathway” in WikiPathways (WP) and “YAP1‐ and WWTR1 (TAZ)‐stimulated gene expression” in Reactome, positively enriched in hyper‐ compared to hypo‐osmotic condition (Figure 5B,C). Therefore, we speculated that YAP, which is a major downstream effector of the Hippo pathway,^[^ ^35^ ^]^ is involved in various mechanotransduction phenotypes across multiple cell types,^[^ ^36^ ^]^ and might potentially be a key central transcription factor for the barrier‐protecting effect against vascular inflammation. Since YAP, along with its homolog TAZ, is known to localize to the cell nucleus upon activation,^[^ ^35^ ^]^ we investigated whether hyperosmolarity‐adapted cells display distinct YAP localization patterns. Although the overall YAP intensity was lower than hypo‐ and iso‐osmolarity adapted microvessels, hyperosmolarity‐adapted microvessels displayed a significant increase in YAP nuclear localization, which was verified by both immunofluorescence imaging (Figure 5D,E, and Figure S20, Supporting Information) and western blot (Figure 5F). This is consistent with a previous report that hyperosmotic exposure opens the nuclear pores for YAP entry.^[^ ^37^ ^]^ We then checked if YAP is indeed required for the osmolarity‐induced barrier function modulation. When YAP activity was inhibited by siRNA (Figure S19I, Supporting Information), microvessels adapted to all osmotic conditions displayed significantly increased 4 kDa FITC‐dextran leakage (Figure 5G), indicating low barrier function (Figure 5H). Based on these results, we hypothesized that osmolarity‐driven YAP regulation drives mechanoprotection of microvessels under vascular inflammation.

**Figure 6:**
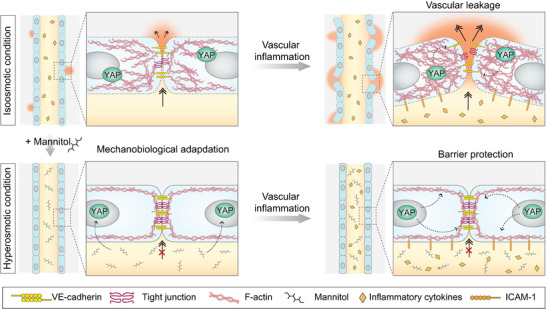
Proposed mechanism of the Yes‐associated protein (YAP)‐mediated mechanoprotective effect of hyperosmolarity in engineered human microvessels.

YAP nuclear localization is considered irreversible, subsequently storing past mechanical memory and driving mechanical changes even after the stimuli vanishes.^[^ ^38^ ^]^ Thus, we questioned if our hyperosmolarity‐induced barrier enhancement can be maintained even after the osmolarity recovers to iso‐osmolarity, mimicking the homeostasis of body fluids after osmotherapy.^[^ ^13^ ^]^ To test this, we assessed the barrier functions of the hyper‐ and hypo‐osmolarity‐adapted microvessels 24 h after the osmolarity recovered to the iso‐osmotic condition (Figure 5I). The barrier function impairment previously observed in hypo‐osmolarity‐adapted microvessels was rescued following 24 h iso‐osmotic recovery (Figure 5J). However, the hyperosmolarity‐induced improvement in barrier function was sustained for additional 24 h even after hyperosmotic infusion was ceased and recovered to iso‐osmotic conditions (Figure 5J,K). Similar phenotypic changes were observed in the VE‐cadherin intensity levels and localization (Figure 5M,N); cells that were adapted to hypo‐ conditions, which initially display weaker VE‐cadherin intensity, recovered close to the iso‐ conditions soon after the osmolarity was reversed to iso‐osmotic condition. Hyperosmolarity‐adapted cells, on the other hand, continuously displayed intensified VE‐cadherin levels and localization at the cell–cell junction even after iso‐osmotic recovery. Surprisingly, the increase in YAP nuclear localization after 24 h of hyperosmolarity exposure was sustained until 72 h after iso‐osmotic recovery (Figure 5O), suggesting that proactive hyperosmolarity exposure during the early microvessel maturation period may provide long‐term barrier improvements and defense against vascular inflammation (**Figure** **6**).

## 3 Discussion

Our work resolves the seemingly paradoxical effects (i.e., beneficial vs deleterious) of hyperosmolarity on vascular barrier integrity. Numerous studies report that strong and acute hyperosmolarity (>1000 mOsm L^−1^, <10 min) induces sudden shrinkage of brain endothelial cells,^[^ ^39^ ^]^ consequently rupturing the cell–cell junction of the BBB, both in vivo and in vitro.^[^ ^10^, ^40^ ^]^ Consistently, we did not observe improvements in barrier function when hyperosmotic shocks were acutely introduced after the microvessel maturation (Figure S8, Supporting Information). In fact, the beneficial effect of acute hyperosmotic shock has only been reported in rat‐derived endothelial monolayers^[^ ^41^ ^]^ and rat‐excised venules,^[^ ^6^ ^]^ but not yet in human vascular barriers.

In contrast, vascular barrier functions were significantly improved when hyperosmolarity exposure started before the vessel matured (i.e., 2 h after seeding) and continued until the barrier matured (Figure 1). We showed that subsequent adaptation to hyperosmolarity exposure upregulates adherens junction protein VE‐cadherin expression (Figure 2), cortical F‐actin localization to cell–cell junctions, and the junctional tension (Figure 3), indicating that the “time window” of osmotic control is critical in the induction of the vascular barrier‐enhancing effect. We speculate that the exposure to high osmolarity on a short‐time scale may mechanically rupture endothelial cell–cell junction by inducing sudden cell shrinkage, but prolonged exposure to relatively mild hyperosmolarity (<600 mOsm L^−1^) might trigger osmotic stress management and osmoadaptation signaling pathways which consequently leads to improvements in the vascular barrier function. Thus, the key mechanism of how endothelium in the inner renal medulla which is constantly exposed to such high osmolarity^[^ ^42^ ^]^ continues to maintain tight barrier function may lie in the intensity, the timing of their exposure, and duration of adaptation to hyperosmolarity.

Furthermore, our work suggests the potential therapeutic possibility of continuous hyperosmotic exposure during wound healing which may help improve the barrier function of newly generated microvessels. Note that the hyperosmolarity (<600 mOsm L^−1^) implemented in this study is significantly less than the osmolarity of clinically widely used 20% mannitol injection (≈1100 mOsm L^−1^).^[^ ^43^ ^]^ Importantly, the barrier function improvements were sustained for at least an additional 24 h even after the hyperosmotic exposure eventually ceased (Figure 5J,K). Revisiting previous works that have reported beneficial effects of hypertonic solution resuscitation after hemorrhagic shock^[^ ^44^ ^]^ may further narrow down the intensity range and time window of osmotic control to maximize vascular barrier improvements.

The endothelial cell state of the vascular barrier also affects osmolarity‐dependent permeability regulation. The BBB permeability after the sudden hyperosmotic shock was reported to be dependent on the developmental stage of the brains.^[^ ^45^ ^]^ Furthermore, activated endothelial cells with adenosine triphosphate (ATP) 10 × 10^−3^ m showed a decrease in permeability after both hypertonic saline and hypertonic saline dextran,^[^ ^46^ ^]^ whereas non‐activated endothelial cells did not show any significant barrier‐enhancing effect after hypertonic exposure.^[^ ^47^ ^]^ Future studies exploring the relationship between endothelial cell state and hyperosmolarity‐driven barrier function regulation may be helpful in reassessing the efficacy of hyperosmotic solutions in patients with vascular complications.

Importantly, the improvement in barrier function following hyperosmolarity exposure was universally observed in 3D microvasculature‐on‐chip of various human endothelial cell types (Figure 1H–K). The barrier‐enhancing effect following hyperosmotic adaptation might also explain the working mechanism of the hypertonic saline infusion and osmotherapy for patients with ischemic hemorrhage and brain edema associated with ischemic stroke. However, the effect of osmotic stress on brain physiology still remains unclear. Numerous brain vascular chips have been developed to study pathogenic brain infection,^[^ ^21b^ ^]^ neurodegeneration,^[^ ^21^, ^48^ ^]^ and glioblastoma.^[^ ^49^ ^]^ In this regard, osmolarity modulation in the brain vasculature models would be a powerful approach to further elucidate the effect of osmotherapy on brain pathophysiology.

Osmolarity‐dependent modulation in microvessel barrier function has considerable therapeutic potential for the treatment of inflammatory complications, such as sepsis. Compared to the hypo‐ or iso‐osmolarity‐adapted microvessels, hyperosmolarity‐adapted microvessels displayed significantly improved barrier protection against exposure to pro‐inflammatory cytokine (TNF*α*) or mediator (LPS; Figure 4). Several vascular protecting strategies in septic mice have been reported by targeting angiopoietins^[^ ^5^, ^50^ ^]^ and intermedin.^[^ ^5a^ ^]^ Although a few works have reported that hyperosmolarity can suppress inflammation through leukocyte cell shrinking^[^ ^51^ ^]^ and downregulation of pro‐inflammatory cytokine transcriptions,^[^ ^52^ ^]^ hyperosmolarity‐driven vascular protection against inflammation has not been reported elsewhere. In fact, hyperosmolarity‐adapted endothelial cells, while maintaining the vascular barrier tight, showed significantly elevated inflammatory marker ICAM‐1 expression. Therefore, our work uniquely proposes that hyperosmolarity may suppress vascular leakage without compromising the innate immune response of the vascular microphysiological system. In addition, initial hyperosmolarity exposure led to long‐term barrier protection even after iso‐osmotic recovery (Figure 5). Altogether, proactively treating septic patients with hyperosmotic agents may induce long‐lived vascular protection and consequently stop devastating inflammatory cascades in severe sepsis.

In contrast, hyperosmolarity‐adapted microvessels exhibited increased sensitivity toward ROCK and myosin inhibition, displaying elevated vascular leakage upon Y‐27632 or Blebbistatin treatment. Hypo‐ and iso‐osmolarity adapted microvessels did not display a major change following treatment (Figure 3G). Although the ROCK pathway has a dual role regulating vascular integrity,^[^ ^53^ ^]^ a decrease in paracellular leakage (and thus an increase in barrier function) has been reported after treating endothelial monolayers with Y‐27632.^[^ ^54^ ^]^ This barrier‐enhancing effect of Y‐27632 was attributed to the downregulation of cell contractility (i.e., forces pulling the junction toward the cell center). In other studies, monolayer permeability was significantly increased (and thus a decrease in barrier function) following Y‐27632 or Blebbistatin^[^ ^55^ ^]^ treatment. These contradictory results might arise from the geometry and stiffness of the substrate on which the endothelial monolayers are cultured; unlike studies that utilize transendothelial electrical resistance (TEER) and fluorescent permeability assays, in which the monolayers are typically cultured on 2D transwells of stiff substrates, our microvasculature‐on‐chip are engineered onto soft 3D collagens scaffolds. Moreover, the deleterious effect of ROCK on the barrier function through contractile F‐actin stress fibers might be minimal^[^ ^53^ ^]^ in our hyperosmolarity‐adapted microvessels, as the basal level of contractile stress fiber across the cell body is low (Figure S17, Supporting Information). Therefore, we hypothesize that ROCK and its downstream target, myosin, in hyperosmolarity‐adapted microvessels, especially cultured on low stiffness substrate, stabilize the cell–cell junction, rather than disrupting it by contractile pulling forces.

The hyperosmolarity‐adapted microvessels exhibited a significant increase in VE‐cadherin localization to the cell–cell junction compared to the hypo‐ or iso‐osmolarity‐adapted microvessels. The increase in the total levels of VE‐cadherin, rather, was as dramatic as compared to the increase in junctional VE‐cadherin levels (Figure 2). Thus, recruiting/localizing the VE‐cadherins to the cell–cell junction is more prominent than the up‐regulation of total VE‐cadherin expression levels. Mechanistically, actomyosin‐driven membrane tension promotes VE‐cadherin clustering.^[^ ^56^ ^]^ Therefore, an increase in actomyosin‐dependent tension at the adherens junction in hyperosmolarity‐adapted endothelial cells may further recruit VE‐cadherin to the cell–cell junction. Consistently, a previous study reported that submembranous F‐actin assembly increases after hypertonic exposure.^[^ ^57^ ^]^ Rac and Cdc42 were suggested as direct mediators for an increase in actin assembly after hyperosmotic exposure. Interestingly, the crosstalk between the hippo signaling (e.g., YAP/TAZ) and Cdc42/Rac have also been reported in numerous studies.^[^ ^58^ ^]^ Thus, during hyperosmotic adaptation, YAP might i) trigger cortical actin assembly and build tension across the cell–cell junction, ii) recruit and localize VE‐cadherin to the cell–cell junction, and iii) consequently lead to barrier improvements. Exactly through what signaling cascades microvessels mechanically adapt and upregulate their barrier function during hyperosmolarity exposure needs further investigation.

The in vitro 3D vascular engineering platform developed in this study has enabled precise and stable control over the vascular microphysiological environment with improvements in throughput and vascular barrier function quantifications. Yet, the following needs to be addressed in order for our results and platform to be translated to a clinical setting. First, our platform currently oversimplifies the vascular niche. For instance, persistent osmotic stress may induce various leukocytes and epithelial cells’ proinflammatory signaling processes,^[^ ^59^ ^]^ which in turn, affect the vascular barrier function. Circulating blood cells including red blood cells, platelet, and immune cells, may also be affected during the osmotic challenge. Osmotic shocks can induce changes in blood cell deformability,^[^ ^16^ ^]^ which may consequently affect the microvascular blood flow. Hyperosmolarity is known to boost B cell activation and differentiation,^[^ ^60^ ^]^ as well as impairment of blood coagulation, fibrin formation, and platelet function.^[^ ^61^ ^]^ Thus, although hyperosmolarity can promote endothelial barrier function enhancement, its effect on other circulating cells needs to be thoroughly assessed to further determine its overall clinical efficacy. Second, our 3D in vitro vasculatures were cultivated and assessed in the static‐flow condition. Flow‐induced shear stresses are reported to affect the vascular endothelial cell shape, orientation, and even cell–cell junctions.^[^ ^62^ ^]^ Future studies implementing physiological blood flow conditions in the vascular microphysiological system could elucidate how the blood flow affects osmolarity‐driven vascular barrier function regulation. Moreover, we have created the extracellular matrix (ECM) solely based on collagen I. Adopting recently reported co‐culture models of different cell types (i.e., pericytes, astrocytes, fibroblasts) in the surrounding ECM environment^[^ ^23a^ ^]^ may better mimic the in vivo environment. Finally, if patient‐derived cells can be utilized for engineering the patient‐specific vascular microphysiological system, our platform can potentially serve as a personalized therapeutic tool for resolving various microvascular complications.

## 4 Experimental Section

### Cell Cultures

HUVECs (ScienCell, USA), hCMEC/D3 (Cedarlane, Canada), hBMEC (Cell Systems, USA), and hDMEC (Promocell, Germany) were cultured in endothelial cell medium (ScienCell, USA) containing 10% fetal bovine serum (FBS), 1% penicillin‐streptomycin (P/S), and 1% endothelial cell growth supplement (ECGS). Frozen stocks were prepared for all cell types after expanding cells in vendor‐recommended media. Frozen stocks of the following passages were thawed and cultured in T75 culture flasks (Corning, USA), where Passage 1 for all cell type indicates the initial stock received from the vendor; HUVEC: Passage 4, hCMEC/D3: Passage 5–7, hBMEC: Passage 6, hDMEC: Passage 3, 2–3 d prior to cell seeding. All cells were cultured in a humidified 37 °C incubator with 5% CO_2_.

### Osmolarity Perturbations

The osmolarity of the cell culture medium (150–600 mOsm L^−1^) was adjusted by mixing with deionized water or d‐mannitol (Sigma‐Aldrich, USA), d‐sorbitol (Sigma‐Aldrich, USA), and sodium chloride (NaCl, Sigma‐Aldrich, USA) for hypo‐ (150 and 200 mOsm L^−1^) or hyperosmotic (350, 400, 500, 550, and 600 mOsm L^−1^) conditions, respectively. Cell culture media was set to be iso‐osmotic (300 mOsm L^−1^). The hyperosmotic solutions of osmolarity lower than 600 mOsm L^−1^ were prepared by serially mixing 600 mOsm L^−1^ solutions with the cell culture media. Unless specified otherwise in the figure legends, hypo‐ and hyper‐ conditions refer to 150 and 600 mOsm L^−1^, respectively.

### PDMS‐Based Microfluidic Chip Fabrication

The detailed steps for microvessel engineering platform fabrication used in this study were adapted from previous works (29, 30, 33). A 10:1 (w/w) mixture of PDMS (silicon elastomer and curing agent; Sylgard 184, Dow corning, USA) was poured onto the house‐designed aluminum mold with microneedles inserted. Microneedles of 550 and 235 µm in diameter were used to create two side channels and the main channel (i.e., where cells are seeded), respectively. A PDMS‐filled aluminum mold was sandwiched with two thick glasses below and on top to ensure flat surfaces and was cured for 2–3 h at 80 °C. The microneedles were subsequently taken out and the microchannel PDMS layer was separated from the mold. Next, a rectangular hole (i.e., space created in between the glass slide and top flat PDMS layer) of 5 × 10 mm dimension was punched for a later collagen chamber. A flat PDMS layer was then bonded on top of the punched, microchanneled PDMS layer using oxygen plasma (Femto Science Co., South Korea). A total of 18 circular holes (8 mm diameter) were punched per device to create the medium reservoir, and six circular holes (1 mm diameter) were punched at two diagonal vertices of the collagen chamber to create collagen input/output ports. Next, microneedles of the same dimensions were inserted into the integrated PDMS device, and the device was sterilized by soaking in 70% ethyl alcohol (EtOH) for >1 h, followed by 60 °C oven‐dry overnight and subsequent UV irradiation for ≈1 h.

The following changes were integrated into the chip design and fabrication to improve the throughput of microvessel engineering and subsequent image analysis for barrier function quantification: i) three rectangular chambers and medium reservoirs connected to each chamber were positioned in parallel onto the single device, ii) rectangular chamber dimensions were increased to 5 × 10 mm to engineer longer microvessels, and iii) the distance between the main channel and two side channels was slightly increased to minimize the flow across the rectangular chambers.

### 3D Cylindrical Collagen I ECM Scaffold

The integrated PDMS chip described above was then bonded to the cover glass slide (50 × 70 mm, Matsunami Glass Inc., Japan) after oxygen plasma treatment. The rectangular chambers were coated with 2 mg mL^−1^ dopamine hydrochloride dissolved in 10 × 10^−3^ m tris‐HCl buffer (pH 8.5) through the 1 mm circular holes at one of the two vertices of the rectangular chamber for 3–4 h at room temperature (RT). After 3× washes with deionized water (DI) followed by 1× wash with phosphate‐buffered saline (PBS; Lonza, Switzerland), the chamber was then loaded and incubated for 30 min at 37 °C with the 3 mg mL^−1^ collagen type I solution. The collagen solution was prepared as follows: i) 100 µL of 10 × Dulbecco's modified Eagle's medium (DMEM) solution (Sigma‐Aldrich, USA) was mixed with 9 µL of 1 m sodium hydroxide (NaOH; Sigma‐Aldrich, USA), ii) 591 µL of 1× DMEM (Welgene, Korea), and iii) 300 µL of 10 mg mL^−1^ collagen I rat tail solution (Corning, NY) were added, iv) mixed well and 1 m NaOH were further injected until the pH was ≈7.2. All steps were performed on ice to prevent any gelation.

Following collagen gelation, the inserted needles were removed and hollow cylindrical channels were created. The needles were then placed at the edge of the PDMS device to separate each circular medium reservoir and prevent leakage from the device.

To image collagen fibrils shown in Figure S3A (Supporting Information), samples were incubated with 5 × 10^−6^ m 5‐(and‐6)‐carboxyte‐tramethylrhodamine (TAMRA) succinimidyl ester (Invitrogen, USA) in PBS at RT for 1 h, followed by 3× PBS wash. The samples were then resuspended in PBS. All labeling and washing steps were carried out by infusing the solution onto two reservoirs connected to one of the side channels and one reservoir connected to the main channel.

### Cell Seeding and Maintenance

All endothelial cell types described in this study were detached from the culture flask using standard trypsinization. Cell solutions were neutralized with heat‐inactivated FBS (Corning, NY) and the cell culture medium described above. The neutralized cell solution was centrifuged at 300× *g* for 3 min and resuspended in the cell culture medium at a final concentration of 1–1.5 × 10^4^ cells mL^−1^. Immediately after resuspension, the cell solution (10–15 µL) was seeded in the main channel. After 5–10 min, 10–15 µL cells were additionally seeded on the other side of the channel. In addition, to facilitate the cell attachment evenly across the cylindrical, hollow channel in the collagen chamber, chips were occasionally flipped upside‐down during cell seeding. Approximately 15 min after cell seeding on both sides of the channel, 100 µL fresh cell culture medium was infused into the main channel to flush out any unattached cells, and the rest of the circular reservoirs as well. Finally, 2 h after cell seeding, all filled solutions in the reservoirs were aspirated and changed to the osmolarity‐adjusted media.

### 2D and 2.5D Endothelial Cell Monolayer Culture

In this study, 2D samples refer to the endothelial monolayers cultured on 0.1 mg mL^−1^ collagen type I coated cover glass and transwell plates. 2.5D samples in turn refer to the endothelial monolayers cultured on 3 mg mL^−1^ collagen gelated beds. To prevent collagens from detaching from the surfaces, cover glasses and well plates were coated with 2 mg mL^−1^ dopamine hydrochloride dissolved in 10 × 10^−3^ m Tris‐HCl buffer (pH 8.5) for 3–4 h at RT, followed by 2× PBS wash, before collagen gelation. The collagen volume gelated in each well was controlled to achieve the bed thickness of 0.5–1 mm, which is similar to the distance from the bottom of the collagen chamber to the lower edge of the microvessels in our 3D in vitro samples. For both 2D and 2.5D samples, cells were typically loaded with ≈1 × 10^5^ cells cm^−2^ concentration.

### Vascular Permeability and Barrier Function Imaging

The media in all reservoirs were aspirated and the PDMS chip was placed on top of the confocal laser scanning microscope (Zeiss LSM 700, Germany). Next, three vertical positions along each microvessel and associated focus *z*‐positions (i.e., total 3 × 3 = 9 location‐focus pairs) were marked using the bright‐field microscopy and saved in the software. The main channel of each microvessel was then sequentially infused with 15 µL of 4 kDa FITC‐dextran (100 × 10^−6^ m) (Sigma‐Aldrich, USA) solution in ≈5 s intervals. This is because the fluorescence imaging of three vertical positions takes ≈5 s. The osmolarity of the dextran solution was matched to the osmolarity of the media in which the microvessels were cultured. Immediately after the last infusion, green fluorescence images were acquired for 5 min at 30 s intervals. All *x*, *y*, and *z* movements, and fluorescence timepoint imaging were automated by the programmed, motorized stage, and built‐in software, respectively (Figure 1A).

### Vascular Permeability and Barrier Function Quantification

To quantify the paracellular permeability and the barrier function of the 3D in vitro microvessels, the fluorescent images of the 4 kDa FITC‐dextran described above were analyzed using the custom MATLAB codes. The detailed image processing steps are described in Figure S2 (Supporting Information).

When the initially calculated vascular permeability was above the value of 8 × 10^−7^ m s^−1^, the slope fitting range was readjusted to the first 60 s, because the fluorescent leakage quickly filled the entire imaging area, thereby saturating the total fluorescent intensity outside the microvessels. For similar reasons, when initially calculated vascular permeabilities were between 1–8 × 10^−7^ m s^−1^, the slope fitting range was readjusted to the first 120 s. For permeability below 1 × 10^−7^ m s^−1^, the slope fitting range was not adjusted (therefore 0–5 min). The original images for calculating permeability of dextrans across the hollow channel in Figure S3 (Supporting Information) were acquired every second. The data were fitted between 10 and 30 s to avoid the initial period of dyes simply infusing into the hollow lumen. For additional details regarding the basic theories of mass transport across the cylindrical lumen, refer to Note S1 (Supporting Information).

### Transwell Permeability Assay

HUVECs were cultured on collagen type 1 (0.1 mg mL^−1^) coated‐ (for 2D) or 3 mg mL^−1^ collagen bed (for 2.5D; see above for additional details) transwell inserts of 24‐well plates (0.4 µm pore, 37024, SPL Life Science, South Korea) at a concentration of 3 × 10^3^ cells/insert. After 1 d, EC media were changed to desired glucose concentration and cultured for 7 d (until fully confluent). Then, 100 µL of EC media containing 10 µg mL^−1^ TRITC‐dextran (4.4 kDa, Sigma, USA) and 500 µL EC media were introduced to the upper inserts and the lower chamber, respectively. After 1 h incubation at 37 °C, EC media in the lower chamber were transferred to 96‐well black plates, and the fluorescence intensity of TRITC‐dextran was measured using the Synergy HTX Multi‐Mode Microplate Reader (BioTek, USA) at the excitation wavelength of 540 nm and emission wavelength of 600 nm. We assumed that the fluorescence intensity of permeated TRITC‐dextran without any cells in the absence (in 2D) or presence (in 2.5D) of collagen bed corresponds to 100% permeability. The fluorescence intensity of each sample was subtracted by background value without TRITC‐dextran. The percentage of permeated TRITC‐dextran was calculated using the following equation

(2) $$\begin{array}{c} \mathrm{Permeated}\;\mathrm{TRITC}-\mathrm{dextran}\;\left(\%\right)=\left(\frac{{\mathrm{FI}}_{\mathrm{samples}}}{{\mathrm{Average}\;\mathrm{of}\;\mathrm{FI}}_{\mathrm{without}\;\mathrm{EC}}}\right)\times 100\;\left(\%\right) \end{array}$$

### Image Analysis—F‐Actin

F‐actin colocalization results in Figure 3B,C and Figure S17 (Supporting Information) were quantified using the custom MATLAB image analysis code. See Figure S15 (Supporting Information) for detailed steps.

### Image Analysis—YAP Colocalization

YAP colocalization results in Figure 5A,B were quantified using the custom MATLAB image analysis code. See Figure S20 (Supporting Information) for detailed steps.

### Image Analysis—Junction Thickness Analysis

The junction thickness and morphology displayed in Figure S11 (Supporting Information) were analyzed using the built‐in functions in Image J (version 2.0.0‐rc‐69/1.52p) software. See Figure S11 (Supporting Information) for additional details.

### Image Analysis—Cell Dimension Characterization

The deep learning‐based cell segmentation algorithm, Cellpose,^[^ ^63^ ^]^ was used to characterize the cell morphology in both 2.5D and 3D culture conditions. The outline of each cell was segmented with VE‐cadherin‐stained image using a cytoplasm filter with a fixed average cell diameter of 40 pixels. The nucleus of the individual cells was segmented with DAPI stained image using a nuclei filter with a fixed average cell diameter of 30 pixels. During the segmentation, the flow threshold and a cell probability threshold were set to 0.4 and 0, respectively. The inaccurately segmented outlines and nuclei of the cells were manually corrected and the corrections were added to the train set for re‐training the algorithm using “Human‐in‐the‐loop.” The final masks for the outline and nucleus of the cells were extracted with the utilization of the re‐trained algorithm.

The shape metrics of the individual cell such as area, perimeter, aspect ratio (major/minor axis length), and circularity were calculated using the ImageJ software. The individual cells were fit to the optimized ellipse produced by Fiji/ImageJ built‐in function for the analysis of the aspect ratio and circularity. To calculate the average cell area in 3D conditions, the half radial surface area (i.e., $\frac{1}{2}\;\mathrm{surface}\;\mathrm{area}=\pi rh$, where *r* and *h* denote the radius and height of the vessel, respectively) was divided by the total number of nuclei observed in the region of interest. Similar to the 2.5D culture condition, inaccurately segmented outlines and nuclei of the cells were manually corrected. See Figure S12 (Supporting Information) for the graphical illustration of the workflow.

### Immunofluorescence Imaging

All immunofluorescence images displayed in the figures were prepared as follows: samples were fixed with 4% paraformaldehyde for 10 min (2D and 2.5D) or 20 min (3D) at RT; fixatives were aspirated and the samples permeabilized with 0.2% Triton X‐100 in PBS for 10 min (2D and 2.5D) or 20 min (3D); for 3D samples, all labeling and washing steps were carried out by infusing the solution onto two reservoirs connected to one of the side channels and one reservoir connected to the main channel. In this way, the remaining solutions in the channels and the collagen chamber from the previous steps were washed away. 3D samples were fixed and permeabilized for an additional 10 min due to the relatively slow infusion process of the solutions across the collagen chambers. Following 2× PBS washes, the samples were incubated with 1% BSA in PBS for 60 min at RT. The samples were then incubated with primary antibody solutions diluted in 1% BSA in PBS at 4 °C overnight. The following day, samples were washed 2× with PBS, and if needed, the secondary antibody labeling was carried out for 1–2 h at RT. Phalloidin tetramethylrhodamine B isothiocyanate (Phalloidin‐TRITC) was diluted together with the secondary antibodies in 1% BSA in PBS solution. After incubation, cell nuclei were labeled for 10 min (2D and 2.5D) or 20 min (3D) at RT. Finally, the samples were washed at least 2× with PBS and kept in PBS.

The following dilution ratios were used for primary antibodies: VE‐cadherin (1:200), anti‐CD‐31 (1:100), anti‐pMLC2 (1:100), anti‐YAP (1:100), anti‐ZO‐1 (1:50), and anti‐Ki‐67 (1:100). A dilution ratio of 1:1000 was used for all secondary antibodies (i.e., Alexa Fluor IgG). The following concentration was used for the remaining antibodies: phalloidin‐TRITC (1.5 µg mL^−1^), DAPI (5 µg mL^−1^), Hoechst 33342 (5 µg mL^−1^).

Samples were imaged using a confocal laser scanning microscope (Zeiss LSM 700, Germany). For 2.5D and 3D sample imaging, 20× and 10× objectives were typically used. No binning was used. The image resolution was ≈6.4 and ≈3.2 pixels µm^−1^ in the *x*–*y* plane. For the 3D microvessel imaging, ≈250 µm thick sections from the bottom of the vessel lumen were typically imaged by sequential z‐stacks 4 µm apart. For 2.5D or 2D monolayer imaging, ≈15–20 µm thick sections from the bottom of the monolayer were typically imaged by sequential z‐stacks 1 µm apart. The displayed projection images were generated by the maximum intensity projection of acquired z‐stacks.

### Antibodies and Chemicals

Anti‐VE‐cadherin (sc‐9989, Santa Cruz, CA), anti‐ZO‐1 (33‐9100, Invitrogen, USA), anti‐phospho‐Myosin Light Chain 2 (pMLC2, #3671, Cell Signaling Technology, USA), and anti‐YAP (#14074, Cell Signaling Technology, USA) were used for immunofluorescence staining and western blotting.

Anti‐ICAM‐1 (sc‐390483, Santa Cruz, CA), ROCK1 (sc‐17794, Santa Cruz, CA), F‐actin (MA1‐80729, Invitrogen, USA), anti‐Myosin Light Chain 2 (MLC2, #3672, Cell Signaling Technology, USA), anti‐ß‐Actin (sc‐47778, Santa Cruz, CA), anti‐GAPDH (sc‐47724, Santa Cruz, CA), anti‐Lamin A/C (sc‐7292, Santa Cruz, CA), anti‐ß‐tubulin (sc‐5274, Santa Cruz, CA), horseradish peroxidase (HRP)‐linked anti‐rabbit IgG (7074, Cell Signaling Technology, USA), HRP‐linked anti‐mouse IgG (#7076, Cell Signaling Technology, USA), and HRP‐linked anti‐mouse IgM (31440, Invitrogen, USA) antibodies were used for western blotting.

Phalloidin‐TRITC (P1951, Sigma‐Aldrich, USA), anti‐VE‐cadherin, Alexa Fluor 647 conjugated (sc9989 AF647, Santa Cruz, CA), anti‐CD31 (ab32457, Abcam, UK), anti‐Ki‐67 (#9129, Cell Signaling Technology, USA), DAPI (D9564, Sigma‐Aldrich, USA), Alexa Fluor 488 goat anti‐mouse IgG (A‐11001, Invitrogen, USA), Alexa Fluor 488 goat anti‐rabbit IgG (A11008, Invitrogen, USA), Alexa Fluor 594 goat anti‐rabbit IgG (A‐11012, Invitrogen, USA), and Alexa Fluor 647 goat anti‐rabbit IgG (A‐21244, Invitrogen, USA) were used for immunofluorescence staining.

(S)‐(‐)‐Blebbistatin (1852, Tocris, USA), Y‐27632 dihydrochloride (1254, Tocris, USA), Latrunculin B (3974, Tocris, USA), Recombinant Human TNF‐alpha Protein (210‐TA, R&D systems, USA), lipopolysaccharides from *Escherichia coli* O111:B4 (LPS; L2630, Sigma‐Aldrich, USA) were used for microvessel chemical perturbation.

### Cell Viability and Proliferation Assays

To assess endothelial cell viabilities in the 3D in vitro microvessels 2 d after osmotic adjustments (Figure S1A, Supporting Information), the live/dead cell staining was first adapted using Calcein AM, cell‐permeant dye (C1430, Thermofisher, USA), and propidium iodide (PI, P3566, Thermofisher, USA). The microvessel lumen and the outer surfaces were infused with 1 × 10^−6^ m Calcein‐AM, 1 µg mL^−1^ PI, and 10 µg mL^−1^ Hoechst 33342 by loading the staining solution in the main and side channels. Cells were incubated with the staining solution for 30 min at 37 °C, followed by a quick wash with cell culture media as described above. Green and red cells indicated live and dead cells, respectively. Cell nuclei were then counterstained with Hoechst 33342.

The viability of endothelial cells in a 2.5D collagen bed was analyzed using the cell proliferation WST‐1 reagent (Roche, Switzerland). HUVEC 2.5D monolayers were incubated with WST‐1 solution for 30 min in a humidified 37 °C incubator according to the manufacturer's protocols. After incubation, the absorbances (*A*) were measured at 450 nm using the Synergy HTX Multi‐Mode Microplate Reader (BioTek, USA). Absorbance values (*A*) were corrected by subtracting the background without WST‐1 solution. The percentage of cell viability was calculated using the following equation

(3) $$\%\;\mathrm{Viability}=\left(A_{\mathrm{sample}}/A_{\mathrm{average}\;\mathrm{of}\;\mathrm{controls}}\right)\times 100$$

The proliferation 3D in vitro microvessels after osmotic adjustments was qualitatively assessed in two independent ways; first, 3D in vitro microvessels at D2 were fixed and stained with anti‐Ki‐67 antibodies (Figure S1C, Supporting Information), which mark proliferating cells but not the G0 quiescent cells. Second, immediately after the osmolarity adjustment, the live cells were incubated with the EdU labeling solution containing cell culture medium for an additional 16 h (i.e., 2–18 h after cell seeding) in a humidified 37 °C incubator with 5% CO_2_. After incubation, cells were fixed and stained with the EdU detection solution (Figure S1D, Supporting Information), which is described in detail in the Click‐iT EdU Cell Proliferation Kit (C10337, Thermo Fisher, USA) user manual.

### Construction of CDH5‐ and YAP1‐Depleted HUVEC

CDH5 siRNA (sc‐36814, Santa Cruz, CA) was used to generate CDH5 (VE‐cadherin)‐depleted HUVEC following the manufacturer's protocol. siRNA targeting YAP1 were purchased from Bioneer (South Korea) with sequences as follows: 5′‐AGGUACUUCCUCAAUCACA dTdT‐3′ with a deoxythymidine dinucleotide overhang (siYAP). 1 µg of CDH5 siRNA (siCDH5), YAP1 siRNA (siYAP), and RNAiMAX (Invitrogen, USA) were diluted in optiMEM (Invitrogen, USA), and gently added onto cultured cells and incubated for 24–48 h at 37 °C. Following incubation, target genes‐depleted HUVECs were seeded on a 2.5D collagen bed and/or chip under hypo‐, iso‐, and hyperosmotic conditions for an additional 48 h.

### Western Blotting

HUVEC total proteins on the chip and 2.5D collagen bed were extracted using RIPA cell lysis buffer (1×, GenDepot, USA) supplemented with Halt protease and phosphatase inhibitor cocktail (Thermo Scientific, USA). Nucleus and cytoplasmic fractionation were performed using NE‐PER nuclear and cytoplasmic extraction reagents (Thermo Scientific, USA) following the manufacturer's manual. The concentrations of extracted proteins were measured using the Bradford assay (Bio‐Rad Protein assay dye reagent concentrate, USA). Exactly 10 µg of protein was used for sodium dodecyl sulfate‐polyacrylamide gel electrophoresis (SDS‐PAGE), then transferred to NitroPure nitrocellulose transfer membrane (LC7033‐300, GenDepot, USA) for blotting. Primary antibodies and HRP‐conjugated secondary antibodies were used to label target proteins. Protein expression was detected using West‐Q Pico Dura ECL solution (W3653, GenDepot, USA) and membranes were imaged using iBright CL750 Imaging System (A44116, Invitrogen, USA).

Expressions of VE‐cadherin and F‐actin protein were measured using ImageJ. VE‐cadherin and F‐actin band signals were normalized to the loading control signal. For better comparison, the normalized values were calculated as fold changes relative to the iso‐osmotic control.

### RNA Isolation for RNA‐Sequencing

Total RNA was isolated using QIAzol lysis reagent (79306, Qiagen, Germany). RNA quality was assessed by Agilent 2100 bioanalyzer (Agilent Technologies, Amstelveen, The Netherlands), and RNA quantification was performed using an ND‐2000 Spectrophotometer (Thermo Fisher, DE, USA).

### Library Preparation and Sequencing

Libraries were prepared from total RNA using the NEBNext Ultra II directional RNA‐Seq kit (NEW ENGLAND BioLabs, Inc., UK). mRNA isolation was performed using the Poly(A) RNA Selection Kit (LEXOGEN, Inc., Austria). The isolated mRNAs were used for the cDNA synthesis and shearing, following the manufacturers’ instructions. Indexing was performed using the Illumina indexes 1–12. The enrichment step was carried out using PCR. Subsequently, libraries were checked using the TapeStation HS D1000 screen tape (Agilent Technologies, Amstelveen, Netherlands) to evaluate the mean fragment size. Quantification was performed using the library quantification kit using a StepOne real‐time PCR system (Life Technologies, Inc., USA). High‐throughput sequencing was performed as paired‐end 100 sequencing using NovaSeq 6000 (Illumina, Inc., USA).

### RNA‐seq Data Analysis

Quality control of raw sequencing data was performed using FastQC (https://www.bioinformatics.babraham.ac.uk/projects/fastqc/). Adapter and low‐quality reads (<Q20) were removed using FASTX_Trimmer (http://hannonlab.cshl.edu/fastx_toolkit/) and BBMap (https://sourceforge.net/projects/bbmap/). The trimmed reads were mapped to the reference genome using TopHat.^[^ ^64^ ^]^ The RC (Read Count) data were processed based on Fragments Per kb per Million reads (FPKM) + Geometric normalization method using EdgeR within R. FPKM values were estimated using Cufflinks.^[^ ^65^ ^]^ Each sample was measured twice and normalized.

### Bioinformatics Analysis

The global transcriptomic gene expression profiles of HUVEC under hypo‐, iso‐, and hyperosmotic conditions, as well as with and without TNF*α* treatments, were assayed by using RNA‐sequencing (RNA‐seq). RNA‐seq data were ranked by fold changes (FC) comparing hypo‐, iso‐, and hyperosmotic conditions, as well as with and without TNF*α* treatment.

Gene ontology (GO) term enrichment analysis was analyzed using EnrichR software (http://amp.pharm.mssm.edu/Enrichr/), ranked by the *p*‐value.^[^ ^66^ ^]^ Gene set enrichment analysis (GSEA)^[^ ^67^ ^]^ was performed using the Molecular Signatures Database (MSigDB version 7.5.1).^[^ ^68^ ^]^ Heatmap visualization is represented by *z*‐scores, calculated by the following equation

(4) $$z\;\mathrm{score}=\left(\mathrm{expression}\;\mathrm{value}-\mathrm{mean}\right)/\mathrm{standard}\;\mathrm{deviation}$$

In Figure 2, over twofold upregulated genes in hyper‐ and iso‐ compared to hypo‐osmotic conditions (Hyper/Hypo > 2 and Iso/Hypo > 2) genes were sorted by GO analysis. GSEA was performed by comparing iso‐ and hypo‐osmotic conditions (Hypo < Iso).

In Figure S14 (Supporting Information), over 1.5‐fold upregulated genes in hypo‐ compared to hyper‐ and iso‐osmotic conditions (Hypo/Hyper > 1.5 and Hypo/Iso > 1.5) were sorted by GO analysis. Heatmaps were visualized by *z*‐scores in the ascending order of FC values of Hypo/Hyper.

In Figure 5, and Figure S19 (Supporting Information), 1.5‐fold upregulated genes in hyper‐ and iso‐ compared to hypo‐conditions (Hyper/Hypo > 1.5 and Iso/Hypo > 1.5), and genes that are maintained after TNF*α* treatment in hyper‐ but not in hypo‐osmotic conditions ([Hyper/HyperTNF*α*] > [Hypo/Hypo TNF*α*]) were sorted by GO analysis.

### Statistical Analysis

The ΔBarrier function displayed after Figure 1H reflects the change in barrier function of each microvessel relative to the mean barrier function of iso‐osmotic control microvessels in each independent set of experiments. ΔBarrier (before − after drug) indicates the change in barrier function of each microvessel after the chemical perturbation.

The number of experimental replicates, statistical methods, and significances are specified in each figure caption. In RNA‐seq analysis, one sample in each condition was analyzed, and the representative value normalized through two measurements was used for bioinformatic analysis. Typically, statistical comparisons within a single group were carried out using one‐sample, two‐sided *t*‐test compared to the value of 0. Statistical comparisons between two experimental groups were performed using an unpaired, two‐tailed *t*‐test, and comparisons among more than three groups were performed using one‐way analysis of variation (ANOVA), followed by Tukey's test for post hoc analysis. *P*‐values are represented with asterisks (*) as follows; *p < 0.05; **p < 0.01; ***p < 0.001; ****p < 0.0001. All experiments were repeated at least three times. MATLAB (Mathworks, USA) and Prism ver. 9 (GraphPad, USA) software were used for the statistical analysis.

## Conflict of Interest

The authors declare no conflict of interest.

## Author Contributions

J.H.K. and M.J. contributed equally to this work. J.H.K., M.J., S.J.S., and S.S. designed and performed the experiments. J.H.K., A.C., and D.S. carried out image analysis. M.J. performed immunoblot and RNA‐sequencing analysis. J.H.K., S.H.L., and H.N.K. supervised the work. J.H.K. and M.J. drafted the manuscript with input from all authors. J.H.K. conceived and initiated the study.

## Supporting information

Supporting Information

## Data Availability

The data that support the findings of this study are available from the corresponding author upon reasonable request.
