# Supplementary material for: Mechanobiological Adaptation to Hyperosmolarity Enhances Barrier Function in Human Vascular Microphysiological System
Source: Adv Sci (Weinh). 2023 Feb 19;10(13):2206384. doi: 10.1002/advs.202206384 (PMC10161024; doi:10.1002/advs.202206384)
Supplement: Supplementary file 1 — Supporting Information [file ADVS-10-2206384-s001.pdf]

ADVANCED SCIENCE
Open Access

Supporting Information

for *Adv. Sci.*, DOI 10.1002/advs.202206384

Mechanobiological Adaptation to Hyperosmolarity Enhances Barrier Function in Human Vascular Microphysiological System

Joon Ho Kang, Minjeong Jang, Su Jin Seo, Andrew Choi, Daeeun Shin, Suyoung Seo, Soo Hyun Lee* and Hong Nam Kim**

Supporting Information

Mechanobiological adaptation to hyperosmolarity enhances barrier function in human vascular microphysiolgical system

Joon Ho Kang[+*], Minjeong Jang[+], Su Jin Seo, Andrew Choi, Daeeun Shin, Suyoung Seo, Soo Hyun Lee[*], Hong Nam Kim[*]

J. H. Kang, M. Jang, S. J. Seo, A. Choi, D. Shin, S. Seo, S. H. Lee, H. N. Kim
Brain Science Institute, Korea Institute of Science and Technology, Seoul 02792, Republic of Korea
* Email: jhkang@kist.re.kr, shleekist@kist.re.kr, and hongnam.kim@kist.re.kr
[†]These authors contributed equally to this work.

S. J. Seo
Department of Chemical Engineering, Kwangwoon University, Seoul 01897, Republic of Korea

D. Shin
School of Mechanical Engineering, Sungkyunkwan University, Suwon 16419, Republic of Korea

S. Seo
Program in Nano Science and Technology, Graduate School of Convergence Science and Technology, Seoul National University, Seoul 08826, Republic of Korea

S. H. Lee, H. N. Kim
Division of Bio-Medical Science & Technology, KIST School, University of Science and Technology (UST), Seoul 02792, Republic of Korea

H. N. Kim
School of Mechanical Engineering, Yonsei University, Seoul 03722, Republic of Korea.
Yonsei-KIST Convergence Research Institute, Yonsei University, Seoul 03722, Republic of Korea.

Supplementary Note I

Theory for calculating flux and permeability across the cylindrical membrane

In this note, we provide a simple theory for calculating the permeability of 3D engineered microvessels from the sequentially obtained FITC-dextran leakage images (Fig. 1a, SFig. 2). The flux of dextran molecules through the cylindrical surface, J, defined by number of dextran molecules passing the membrane of unit area in each second, is given by

$$J = P(c_0 - c_i) \tag{1}$$

where c_0, and c_i are the concentration of molecules inside and outside of the cylinder, respectively. P refers to the permeability of the cylindrical membrane.

The total number of molecules that crossed the lumen at time t, $N(t)$, will be given by

$$N(t) = 2\pi R_0 L \cdot J \cdot t \tag{2}$$

where R_0 and L are the radius and length of the microvessels, respectively. This should be equal to

$$N(t) = \int_{R_0}^{\infty} c(r) \cdot 2\pi r L dr \tag{3}$$

where $c(r)$ refers to the concentration of the dextran molecules at distnace r from the center of the cylindrical lumen, which we can obtain from the average fluorescent intensity plot (SFig. 2c).

Since we typically infuse the lumen with dextran solutions of much larger volume than the lumen (i.e. infinite source; $c_0 = const$), and the space outside the cylinder (i.e. collagen chamber + side channels connected to the reservoirs) is much bigger than the volume inside the lumen (i.e. $c_0 >> c_i \cong 0$), we can approximate our permeate flux to be constant over time. With the following approximation, we get

$$P \cdot t = \int_{R_0}^{\infty} \frac{c(r) r}{c_0 R_0} dr \tag{4}$$

Thus, the permeability of the membrane can be obtained by taking the slope of the line in SFig.2e.

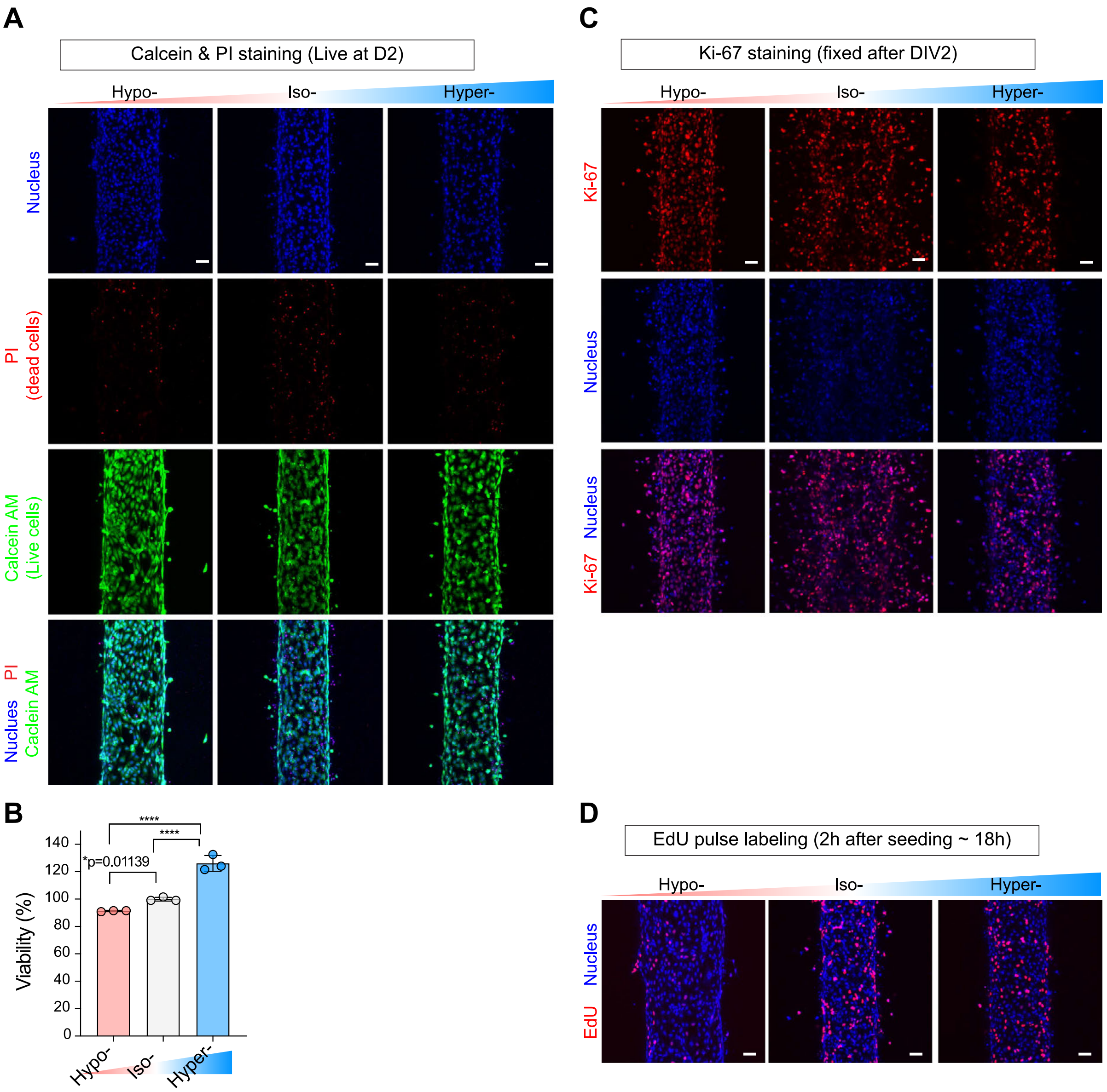

Figure S1. Viability and proliferation after osmotic adaptation.
A) Representative immunostaining of Propidium Iodide (PI; red) and Calcein-AM (green), which stain dead and viable cells, respectively, in live human umbilical vein endothelial cell (HUVEC) 3D engineered microvessels 2 d after corresponding osmotic adjustments (i.e., hypo-, iso-, and hyper-osmotic conditions at D2; see Figure 1B for detailed timelines). Cells were counterstained with Hoechst 33342 (nucleus). **B)** Cell viability of HUVEC 2.5D monolayers after hypo-, iso- and hyper-osmolartiy-adaptation, analyzed by WST-1 assay. Data represents mean ± S.D. n = 3 biological replicates. *P* values were obtained using one-way ANOVA followed by Tukey's HSD post hoc test. n.s: not significant, ****p<0.0001. **C)** Representative immunostaining of Ki-67 in fixed HUVEC 3D engineered microvessels 2 d after osmolarity adaptation. Ki-67 is a widely known proliferation marker. **D)** Representative images of HUVEC 3D engineered microvessels Edu pulse labeled for 16 h immediately after osmotic adjustments (2 h ~ 18 h after seeding). For panels (C and D) cell nuclei were counterstained with 4′,6-diamidino-2-phenylindole (DAPI) (blue). All scale bars, 50 μm.

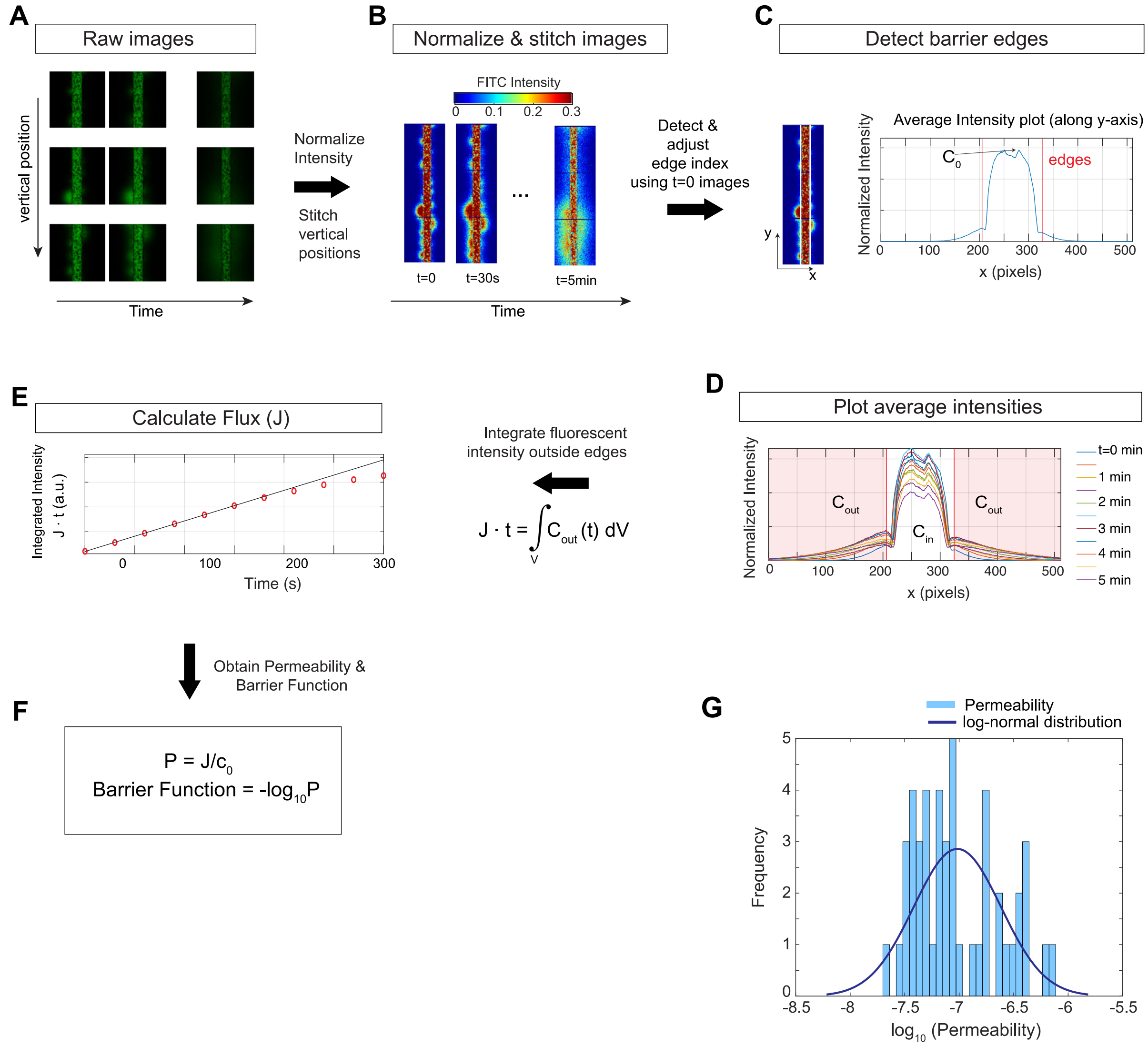

Figure S2. Illustration of paracellular permeability and barrier function calculation steps.
A-G) Successive steps of image analysis and data processing to obtain permeability and barrier function of 3D engineered microvessels. Raw fluorescent images acquired from the confocal microscope (A) were vertically stitched and intensity normalized (B). c_0 indicates the maximum intensity in the t=0 intensity normalized image. The left and right edges of the cylindrical microvessels were detected with the aid of average fluorescent intensity across vertical *y* pixels versus horizontal *x* pixels (C). For each time point, average fluorescent intensities across the vertical y pixels were plotted (D) and the fluorescent intensities outside the edges detected at step described in panel (C) were integrated to obtain the flux (J) through the cylindrical barrier (E). Finally, the paracellular permeability was obtained using the indicated equations (F). Note that the permeability followed an approximately log-normal distribution (G), and thus the new index, barrier function, was defined as a negative logarithm of the permeability.

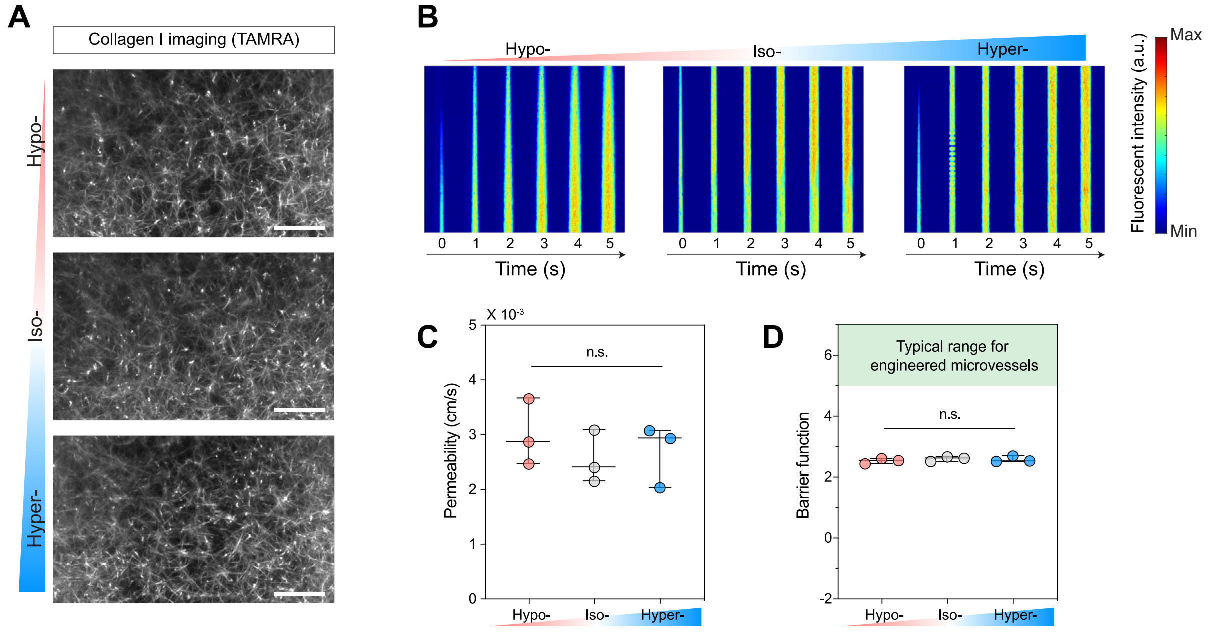

Figure S3. Extracellular matrix (ECM) microstructure and 4 kDa fluorescein isothiocyanate (FITC)-dextran diffusion timescale is not affected by the osmolarity adjustments.

A) Collagen type I fluorescently labeled with 5 µM 5-(and-6)-carboxytetramethylrhodamine (TAMRA), succinimidyl ester *in vitro* 2 d after osmolarity adjustments (hypo-, iso- and hyper-osmolarity). Scale bars, 50 µm. **B)** Representative fluorescent images of osmolarity adjusted 4kDa FITC-dextran leakage from the hollow cylindrical channel (without any endothelial cells). T=0 s images were taken immediately after the hollow lumen was filled with fluorescent dextran. Note that the images were taken every 1 s, rather than 30 s as for microvessel permeability imaging. **C, D)** Permeability (C) and barrier function (D) of the hollow lumen. In panel (D), the green region indicates the typical barrier function ranges of 3D engineered microvessels. Box and whisker plots represent median value (horizontal bars), 25 to 75 percentiles (box edges), and minimum to maximum values (whiskers). n=3 channels per each osmotic condition. Statistical tests were done using one-way ANOVA. n.s. not significant.

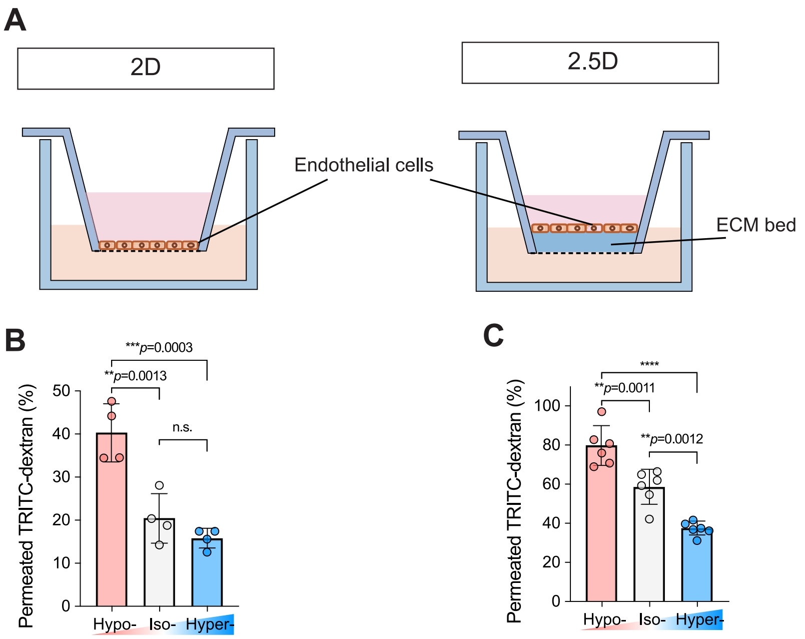

Figure S4. Transwell-based permeability analysis in human umbilical vein endothelial cell (HUVEC) 2D and 2.5D monolayer.

A) Schematic illustration of Transwell-based permeability analysis in HUVEC 2D and 2.5D monolayer. **B, C)** Percentage of permeated TRITC-dextran through HUVEC 2D (B) and 2.5D (C) monolayers after hypo-, iso- and hyper-osmolarity adaptation. Data represents means ± S.D. n = 4 and 6 biological replicates in (B) and (C), respectively. ECM; extracellular matrix. P values were obtained using one-way ANOVA followed by Tukey's HSD post hoc test. n.s: not significant, ****P<0.0001.

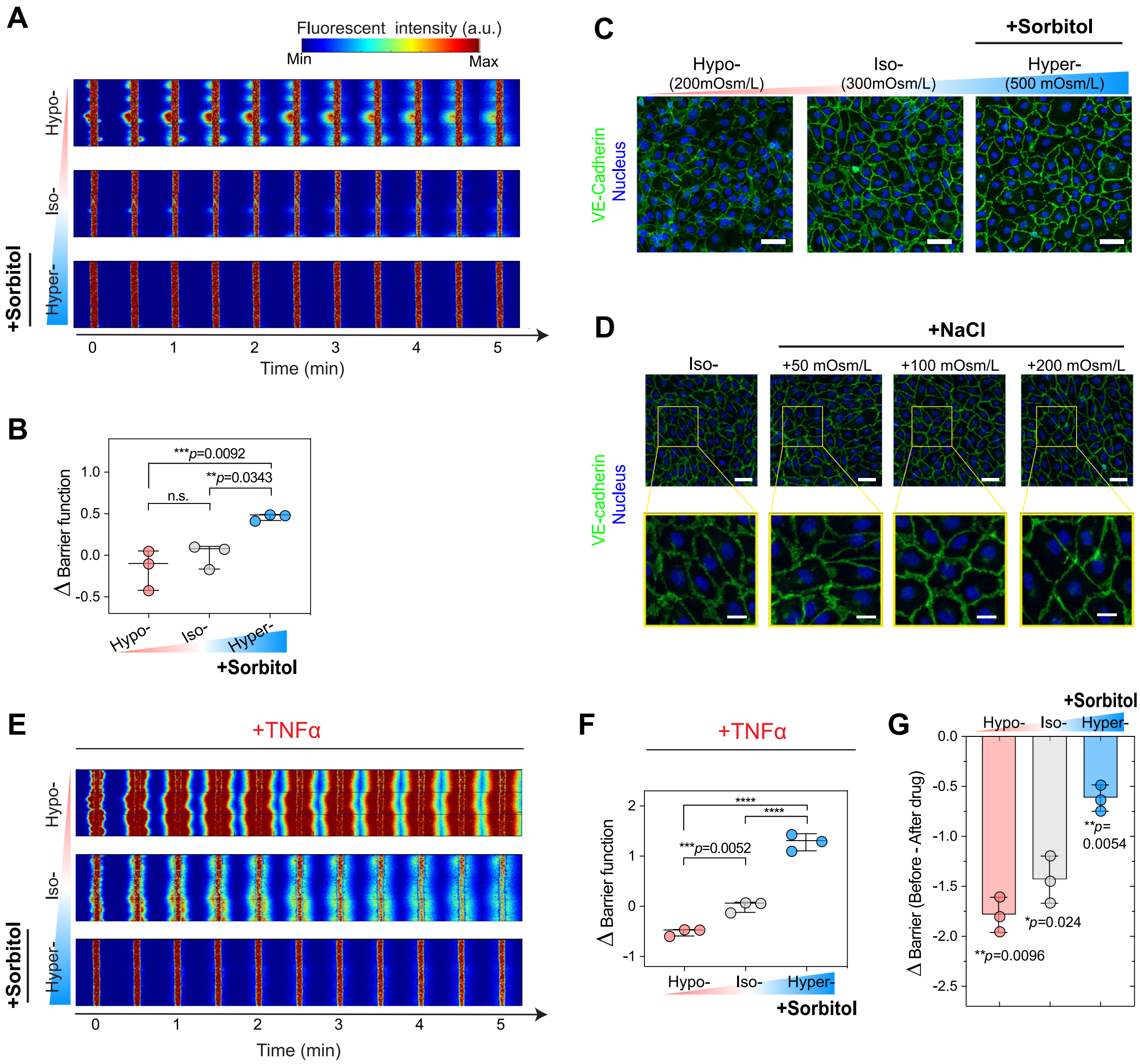

Figure S5. Other hyperosmotic agents produce similar barrier improving phenotypes.
A, B) Representative fluorescent images of 4kDa fluorescein isothiocyanate (FITC)-dextran leakage (A) and barrier function (B) of human umbilical vein endothelial cell (HUVEC) 3D engineered microvessels 2 d after corresponding osmotic adjustments (hypo-, iso-, and hyper-osmotic conditions at D2; see Figure 1B for detailed timelines). Sorbitol was used as a hyperosmotic agent. **C, D)** Immunostaining of VE-cadherin (green) in HUVEC 2D monolayer 2 d after corresponding osmotic adjustments. Sorbitol and sodium chloride (NaCl) was used for creating hyperosmolarity in panel (C) and (D), respectively. Scale bars, 50 µm. Inset: zoom-in view of the yellow regions. Scale bars, 20 µm. **E, F)** Representative fluorescent images of 4kDa FITC-Dextran leakage (E) and barrier function (F) of osmolarity-adapted HUVEC 3D engineered microvessels 24 h after 5 ng/ml TNFα treatment. See Figure 4A for detailed timelines. **G)** Barrier function changes 24 h after 5 ng/ml TNFα treatment. Data represents mean ± S.D. P values obtained by two-tailed, one-sample t-test compared to 0. For panels (A, B, E, F, and G) n=3 microvessels for each osmotic condition. For panels (B and F) box and whisker plots represent median value (horizontal bars), 25 to 75 percentiles (box edges), and minimum to maximum values (whiskers). P values were obtained using one-way ANOVA followed by Tukey's HSD post hoc test. n.s: not significant, ****P<0.0001.

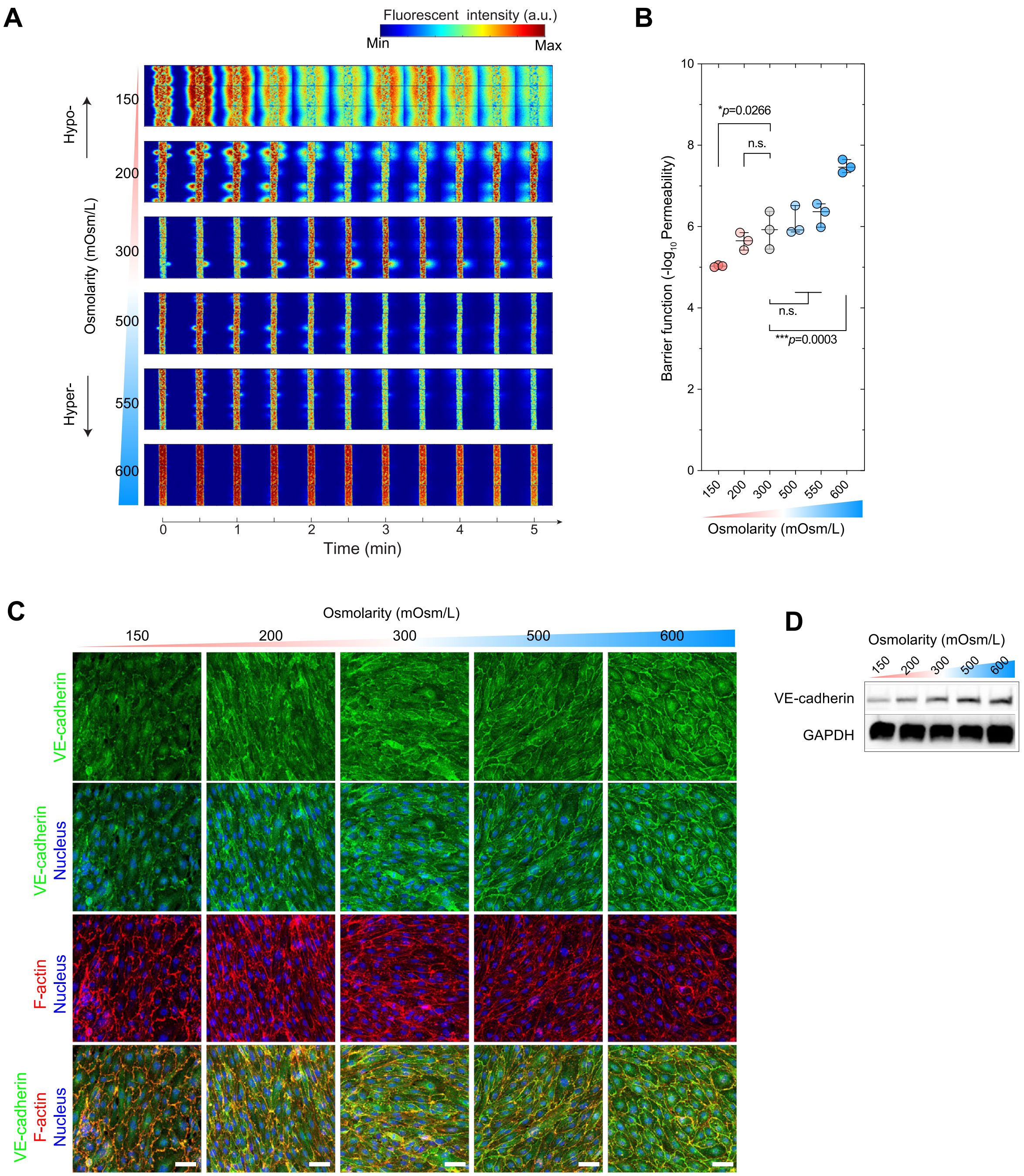

Figure S6. Osmolarity-dependent modulation of the barrier function in extended ranges of osmolarity.
A, B) Representative fluorescent images of 4kDa fluorescein isothiocyanate (FITC)-dextran leakage (A) and barrier function (B) of human umbilical vein endothelial cell (HUVEC) 3D engineered microvessels 2 d after corresponding osmotic adjustments. n=3 microvessels for each osmotic condition. Box and whisker plots represent median value (horizontal bars), 25 to 75 percentiles (box edges), and minimum to maximum values (whiskers). P values were obtained using one-way ANOVA followed by Tukey's HSD post hoc test. n.s: not significant. **C, D)** Immunostaining of VE-cadherin (green) and F-actin (red) (C), and immunoblotting against VE-cadherin (D) in osmolarity-adapted HUVEC 2.5D monolayers. In panel (C) cells were counterstained with DAPI. Scale bars, 50 µm. In panel (D), GAPDH was used as a loading control.

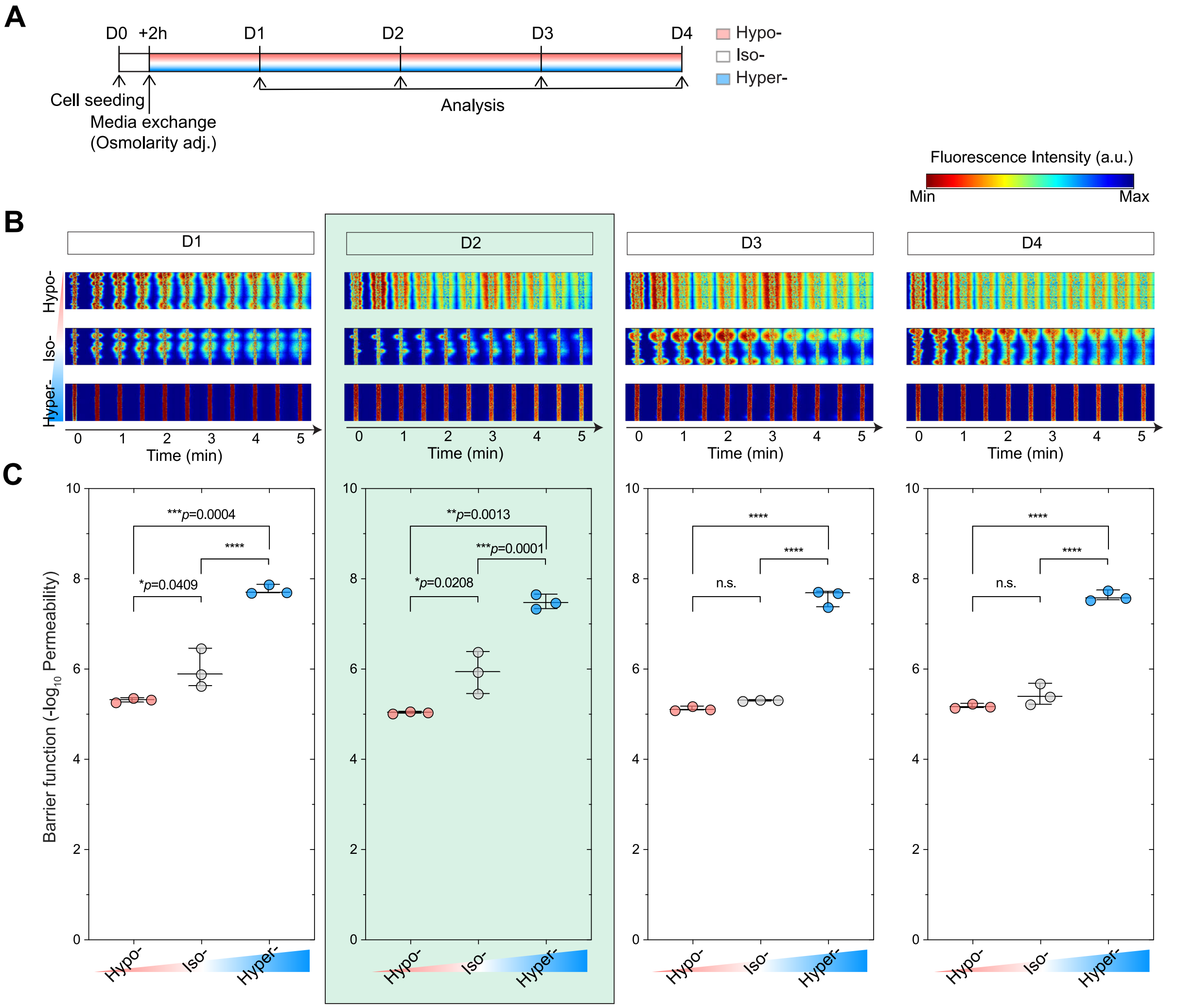

Figure S7. Osmolarity-dependent modulation of the barrier function is maintained for extended periods. **A)** Experimental timeline for testing the effect of long-term exposure to osmotic changes on microvessel barrier function. **B, C)** Representative fluorescent images of 4kDa FITC-dextran leakage (B) and barrier function (C) of osmolarity-adapted (hypo-, iso-, or hyper-osmotic conditions) HUVEC 3D engineered microvessels from D1 to D4. n=3 microvessels for each osmotic condition. Box and whisker plots represent median value (horizontal bars), 25 to 75 percentiles (box edges), and minimum to maximum values (whiskers). P values were obtained using one-way ANOVA followed by Tukey's HSD post hoc test. n.s: not significant. ****P<0.0001. Note that all barrier function quantifications, otherwise noted, were performed at D2.

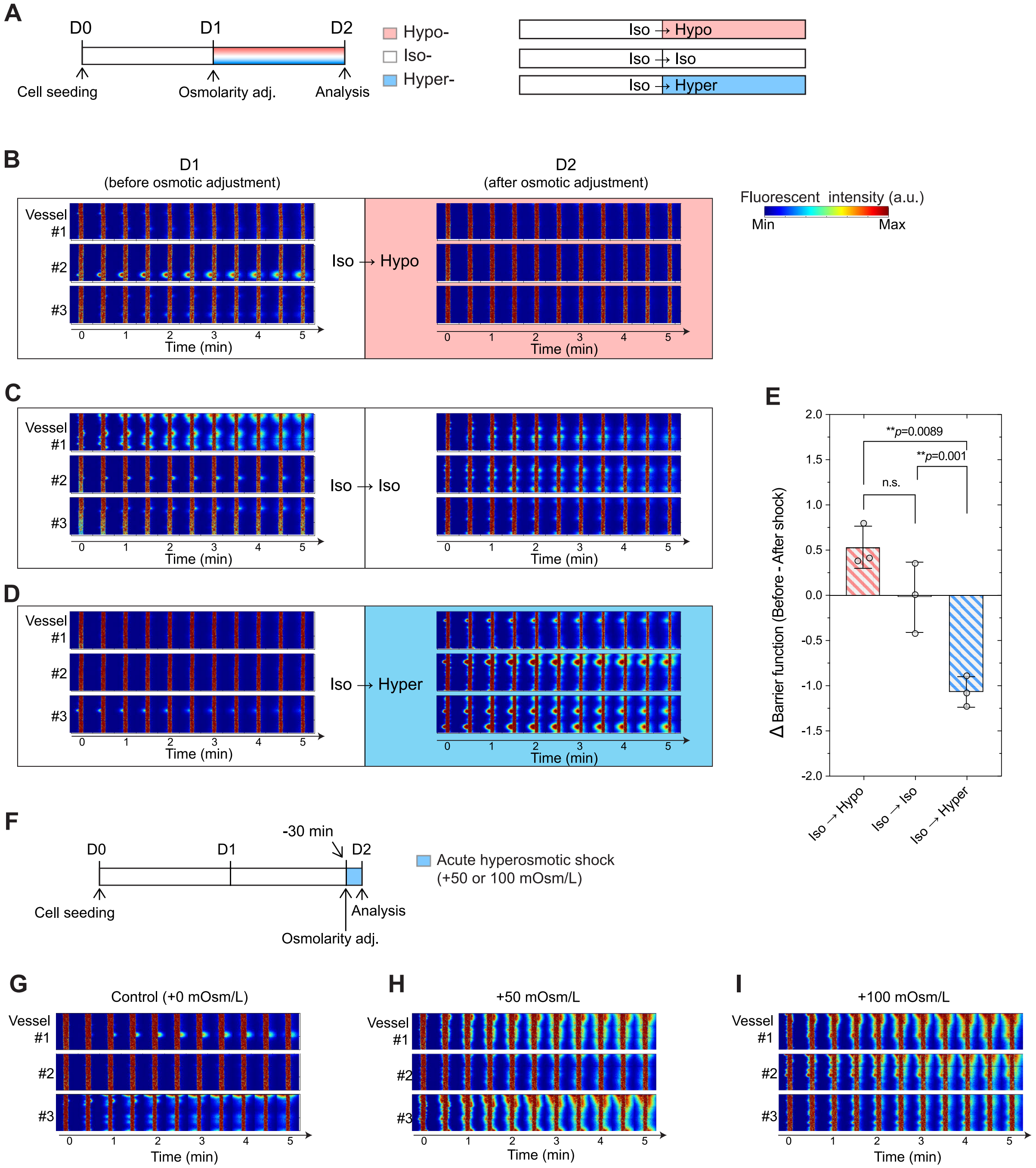

Figure S8. Late or acute hyperosmotic shock after barrier maturation does not improve the barrier function. **A)** Experimental timeline for testing the effect of osmotic exposure on the microvessel barrier function after barrier maturation. **B-D)** Fluorescent images of 4 kDa FITC-dextran leakage from human umbilical vein endothelial cell (HUVEC) 3D engineered microvessels under iso → hypo (B), iso → iso (C), and iso → hyper (D) conditions. **E)** Barrier function change after the osmotic shift at D1. Data represents mean ± S.D. P values were obtained using one-way ANOVA followed by Tukey's HSD post hoc test. n.s: not significant. **F)** Experimental timeline for testing the effect of acute hyperosmotic shock on mature vascular barriers. **G-I)**, Fluorescent images of 4 kDa FITC-dextran leakage from HUVEC 3D engineered microvessels after isoosmotic (G), 350 mOsm/L (H), and 400 mOsm/L (I) media treatments for 30 min.

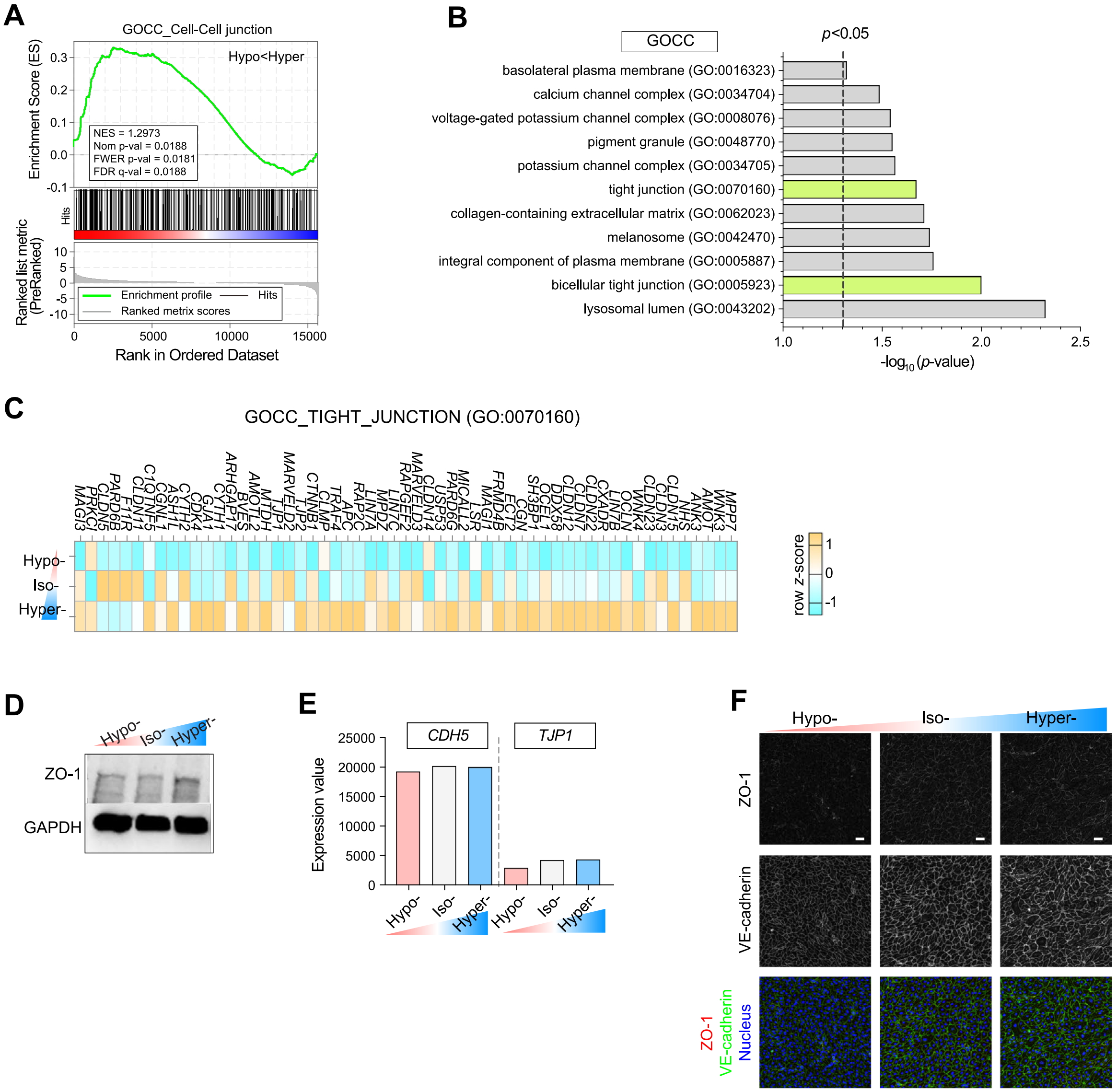

Figure S9. Cell-Cell junction protein expression after osmolarity adaptation.
A) Gene set enrichment analysis (GSEA) results showing significant enrichment of the gene sets, “Cell-Cell junction (GO:0005911)” in GO_cellular components (GOCC) from the Molecular Signatures Database (MSigDB) in hyper- compared to hypo-osmotic conditions. Red and blue shading indicate high and low $\log_2$-ranked values comparing hyper- to hypo-osmotic conditions. NES; normalized enrichment score, NOM p-value; nominal p-value, FWER; familywise-error rate, FDR; false discovery rate. **B)** Gene Ontology (GO) analysis of over 1.2-folds up-regulated 3,061 genes in hyper compared to hypo. The dashed vertical lines indicate significance at $p < 0.05$. Note that gene sets, including ‘bicellular tight junction (GO:0005923)’ and ‘tight junction (GO:0070160)’ in GO_cellular complex were significantly related. **C)** tight junction-related gene sets in GOCC that are upregulated in hyper- compared to iso- or hypo-osmolarity condition. **D)** Expression of ZO-1 protein in HUVEC 2.5D monolayer 2 d after corresponding osmotic adjustments (hypo-, iso-, and hyper-osmotic conditions). GAPDH was used as a loading control. **E)** Comparison of mRNA expression values of *CDH5* (VE-cadherin) and *TJP1* (ZO-1) in osmolarity-adapted HUVEC 2.5D monolayer from RNA-seq results. **F)** Representative immunostaining of ZO-1 and VE-Cadherin in osmolarity-adapted HUVEC 2.5D monolayers. Note that the ZO-1 expression levels in HUVEC cells are significantly lower than VE-cadherin. Cells were counterstained with DAPI. Scale bars, 50 μm.

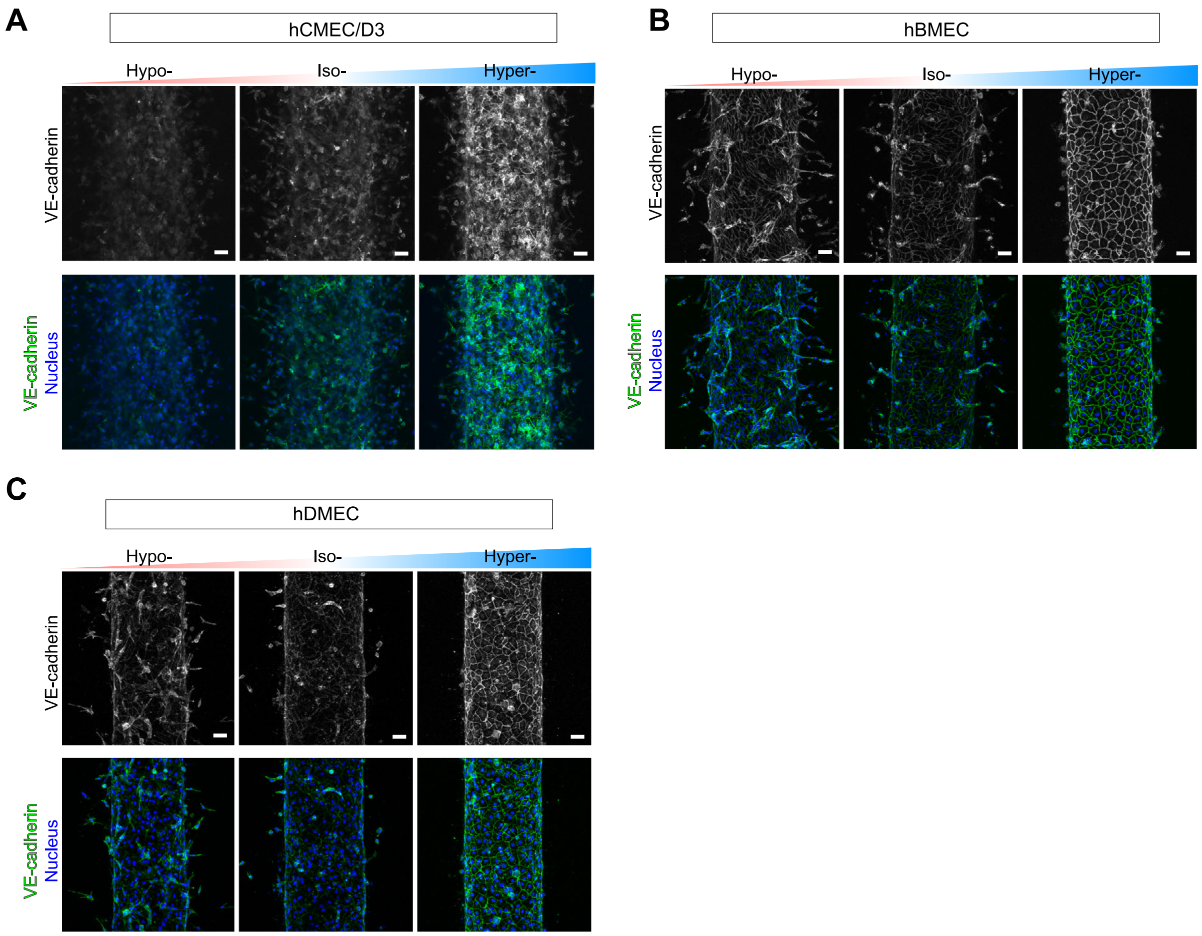

Figure S10. VE-cadherin immunostaining in 3D engineered microvessels of other human endothelial origins. A-C) Representative immunostaining of VE-cadherin in the immortalized human blood-brain barrier cell line, hCMEC/D3 (A), primary human brain microvascular cells, hBMEC (B), and primary human dermal microvascular cells, hDMEC (C) 3D engineered microvessels. Cell nuclei were counterstained with DAPI. Scale bars, 50 µm.

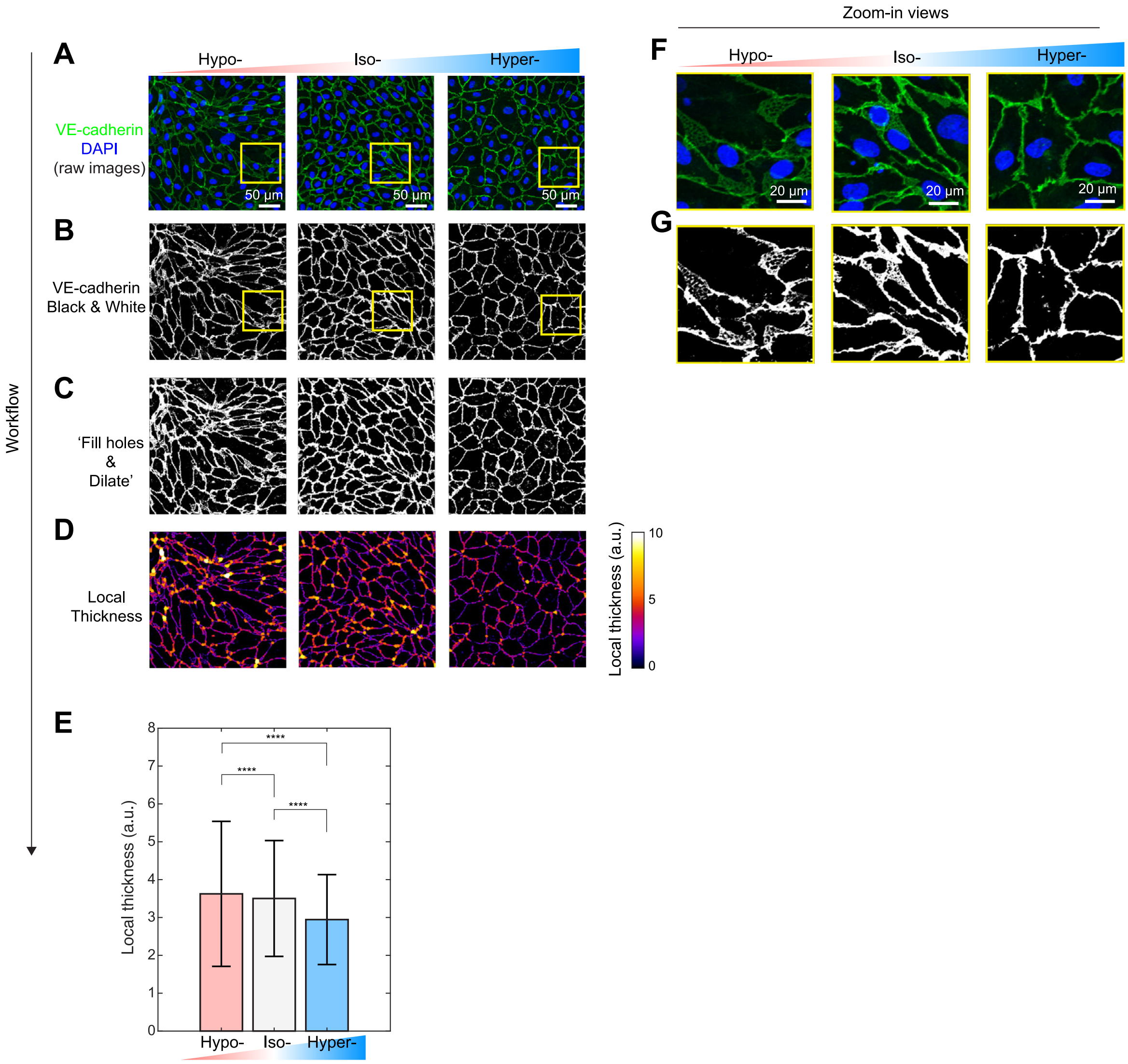

Figure S11. The local adherens junction thickness and morphology changes during osmotic adaptation.
A-E) An illustration of the workflow for quantifying adherens junction thickness. Representative immunostaining of VE-cadherin in the human umbilical vein endothelial cell (HUVEC) 2D monolayer (A). Cells are counterstained with DAPI. Scale bars, 50 μm. The VE-cadherin images are changed to binary black and white images (B). Next, the holes in the junction, especially notable in hypoosmotic conditions, were filled (C). The local thickness of the junction was computed using the ImageJ built-in function (D). Finally, local thickness values of the pixels excluding those with zero local thickness values are plotted (E). Data represent mean ± S.D. n=1144737, 1134877 and 706878 pixels, each from eight cropped images, for hypo-, iso-, and hyper-osmotic conditions, respectively. P values were obtained using one-way ANOVA followed by Tukey's HSD post hoc test. ****$P<0.0001$. **F, G)** Zoom-in views of the yellow regions marked in panel (A) (F) and panel (B) (G). Scale bars, 20 μm.

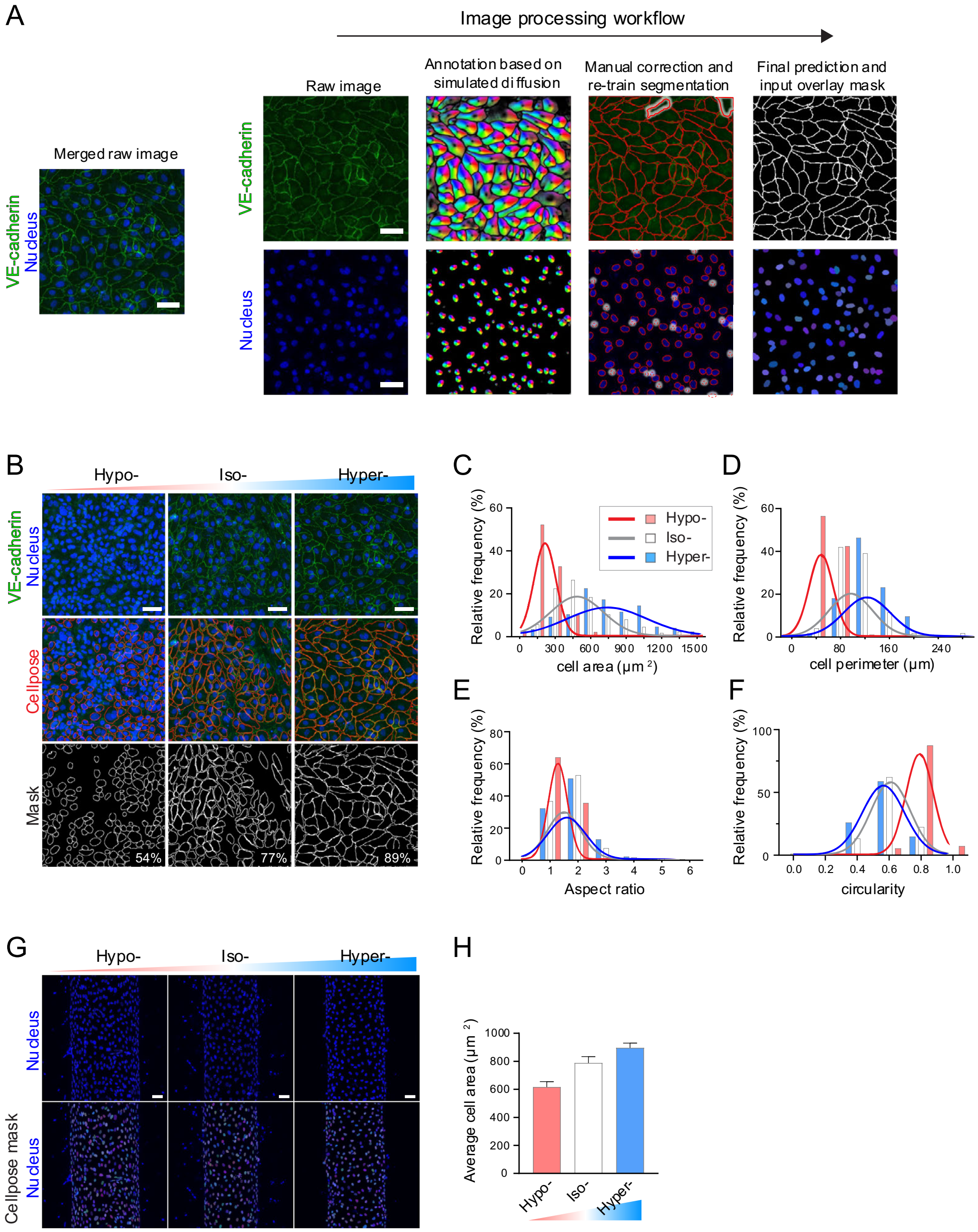

Figure S12. Changes in cell dimensions after osmotic adaptation.

A) Imaging processing workflow for extracting cell outline and nucleus mask from the raw image using Cellpose algorithm. **B)** Representative immunostaining of VE-cadherin (green) and DAPI (blue) in HUVEC 2D monolayer 2 d after corresponding osmotic adjustments (hypo-, iso-, and hyper-osmotic conditions; n = 518, 226 and 200 cells, respectively) and the outlines/mask of individual cell segmented using Cellpose. The percentage of detected cells (white) for different osmotic condition is also indicated in the bottom of each image. Scale bars, 50 µm. **C-F)** The distribution of area (C), perimeter (D), aspect ratio (E), and circularity (F) of segmented individual cells in osmolarity-adapted conditions. Scale bars, 50 µm. **G)** Representative immunostaining of DAPI (blue) in osmolarity-adapted (hypo-, iso-, and hyper-osmotic conditions) HUVEC 3D engineered microvessels and the mask of segmented nucleus. Scale bars, 50 µm. **H)** Average cell area of HUVEC 3D engineered microvessels 2 d after corresponding osmotic adjustments (hypo-, iso-, and hyper-osmotic conditions; n = 7 microvessels for all conditions), which is calculated from the number of cells on the half radial vascular surface. Scale bars, 50 µm.

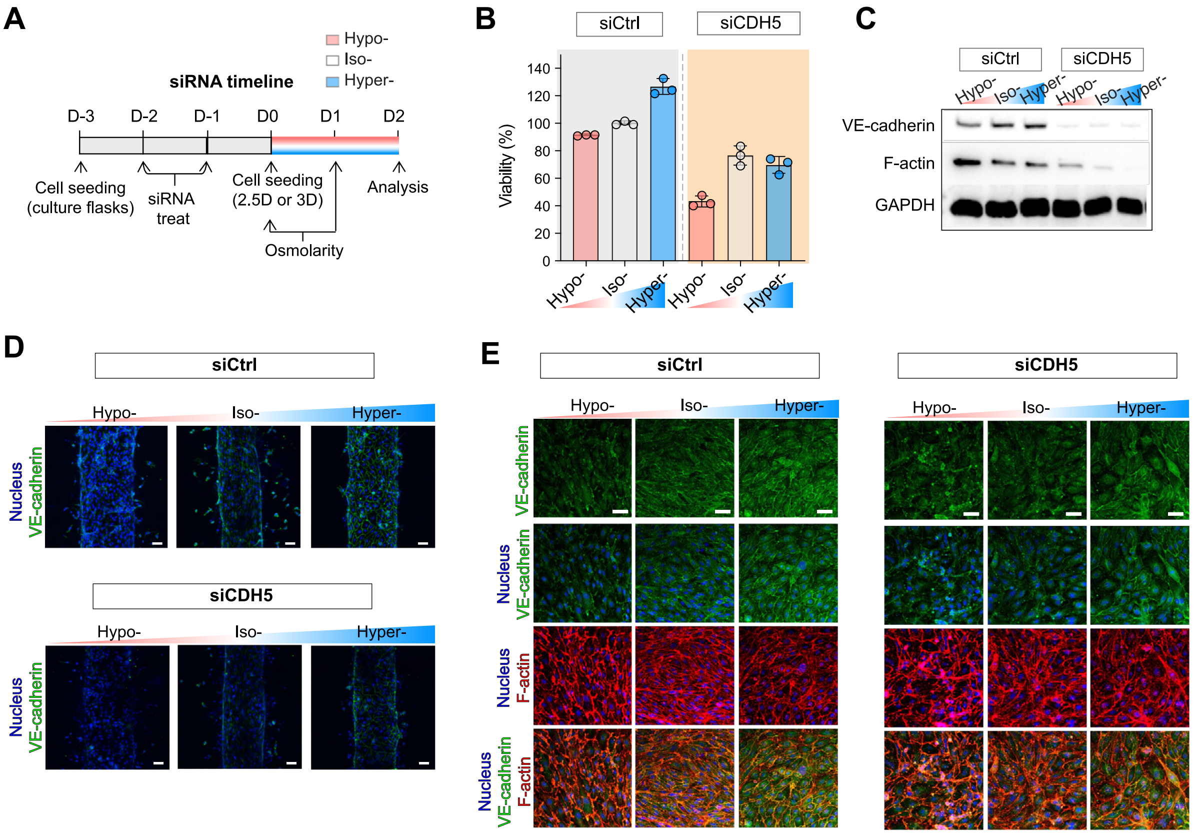

Figure S13. Depletion of VE-cadherin on human umbilical vein endothelial cells (HUVECs) by using small interfering RNA (siRNA) targeting *CDH5* gene (siCDH5).

A) Experimental timeline for treating HUVEC cells with siRNA and testing the effect of osmotic adjustments on siRNA treated HUVEC 3D engineered microvessels or 2.5D monolayers. siRNA is pretreated for 24 h in the culture flasks 2 d before cell seeding. **B)** Viability of siCtrl and siCDH5 treated HUVEC 2.5D monolayers 2 d after corresponding osmotic adjustments (hypo-, iso-, and hyper-osmotic conditions), analyzed by WST-1 assay. Data represents mean ± S.D. n = 3 biological replicates. **C)** Expression of VE-cadherin and F-actin in siCtrl and siCDH5 treated HUVEC 2.5D monolayers after osmolarity adaptation. GAPDH was used as a loading control. **D)** Representative immunostaining of VE-cadherin (green) in siCtrl and siCDH5 treated HUVEC 3D engineered microvessels after osmolarity adaptation. Cell nuclei were counterstained with DAPI. Scale bars, 50 µm. **E)** Representative immunostaining of VE-cadherin (green) and F-actin (red) in siCtrl and siCDH5 treated HUVEC 2.5D monolayer after osmolarity adaptation. Cell nuclei (blue) were counterstained with DAPI. Scale bars, 50 µm.

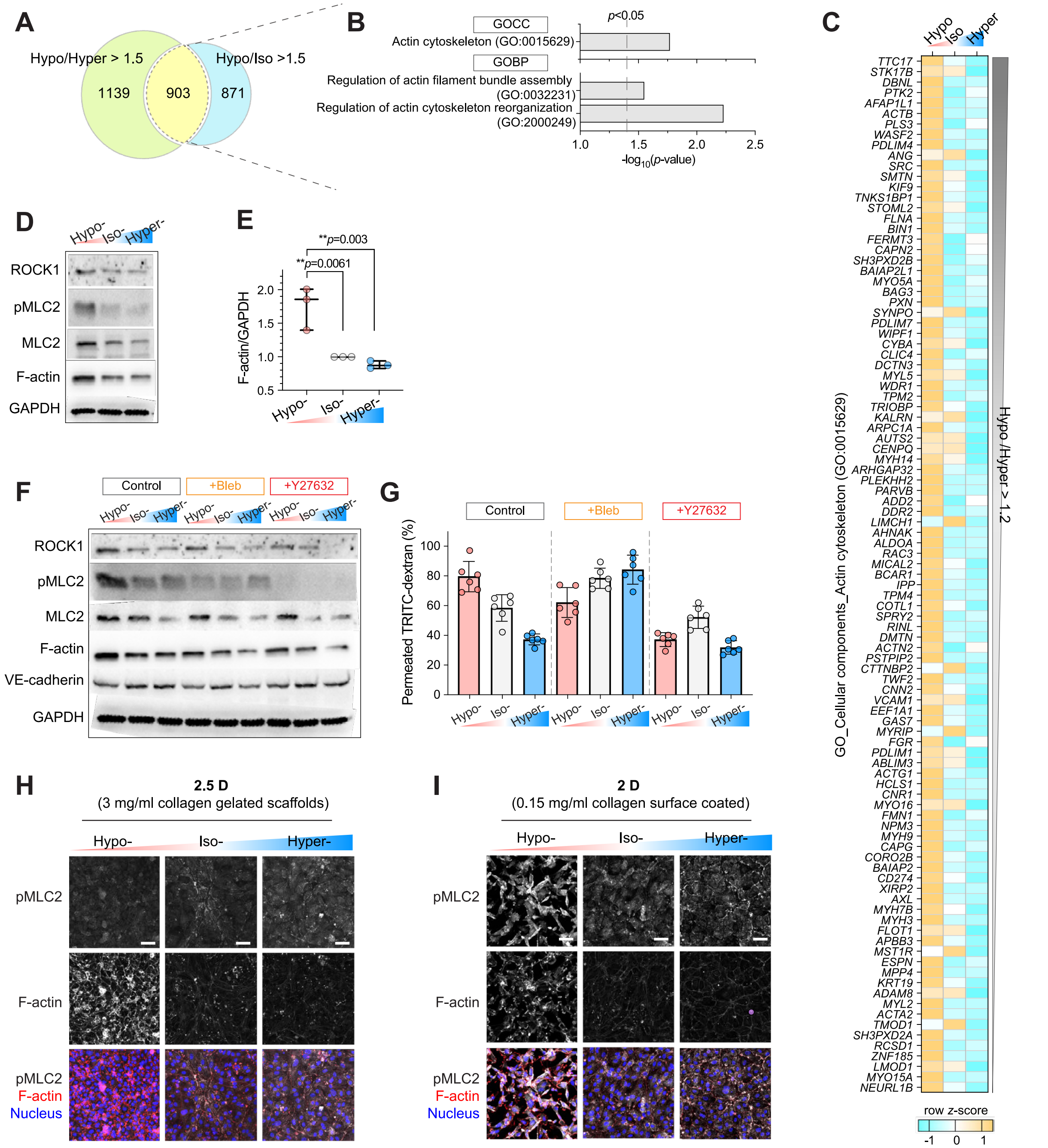

Figure S14. Effect of osmolarity on endothelial cytoskeleton and its related signaling pathway.
A) Number of genes that are upregulated by more than 1.5-folds in hypo- compared to hyper- (Hypo/Hyper > 1.5) and iso-osmotic conditions (Hypo/Iso> 1.5). **B)** Gene Ontology (GO) analysis of the 903 intersected genes. Significantly enriched gene sets were represented from the GO_biological process (GOBP) and GO_cellular components (GOCC). The dashed vertical lines indicate significance at $p < 0.05$. **C)** Heatmap visualization of gene expression profiles of GOCC_actin cytoskeleton (GO:0015629). Genes over 1.2-fold upregulated in hypo- compared to hyper-osmotic conditions are displayed based on the z-score (n = 1). **D)** Expression of cytoskeletal tension-related proteins, ROCK1, pMLC2, MLC, and F-actin in HUVEC 2.5D monolayers 2 d after corresponding osmotic adjustments (hypo-, iso-, and hyper-osmotic conditions). GAPDH used as a loading control. **E)** Western blot-based quantification of F-actin levels relative to isoosmotic conditions. F-actin levels were normalized by GAPDH level. N=3 independent experiments. Box and whisker plots represent median value (horizontal bars), 25 to 75 percentiles (box edges), and

minimum to maximum values (whiskers). P values were obtained using one-way ANOVA followed by Tukey's HSD post hoc test. **F)** Inhibitory effect of 10 μM ROCK inhibitor, Y-27632, and 10 μM myosin II inhibitor, Blebbistatin, on the expression of ROCK1, pMLC2, MLC, F-actin and VE-cadherin in osmolarity-adapted HUVEC 2.5D monolayers. GAPDH used as a loading control. **G)** The percentage of permeated TRITC-dextran through osmolarity-adapted HUVEC 2.5D monolayers 30 min after 10 μM Y-27632 and 10 μM Blebbistatin treatment. Mean ± S.D. n = 6 biological replicates. **H, I)** Representative immunostaining of phosphorylated myosin light chain 2 (pMLC2) and F-actin in osmolarity-adapted HUVEC 2.5D (H) and 2D (I) monolayers. Cells were counterstained with DAPI. Scale bars, 50 μm.

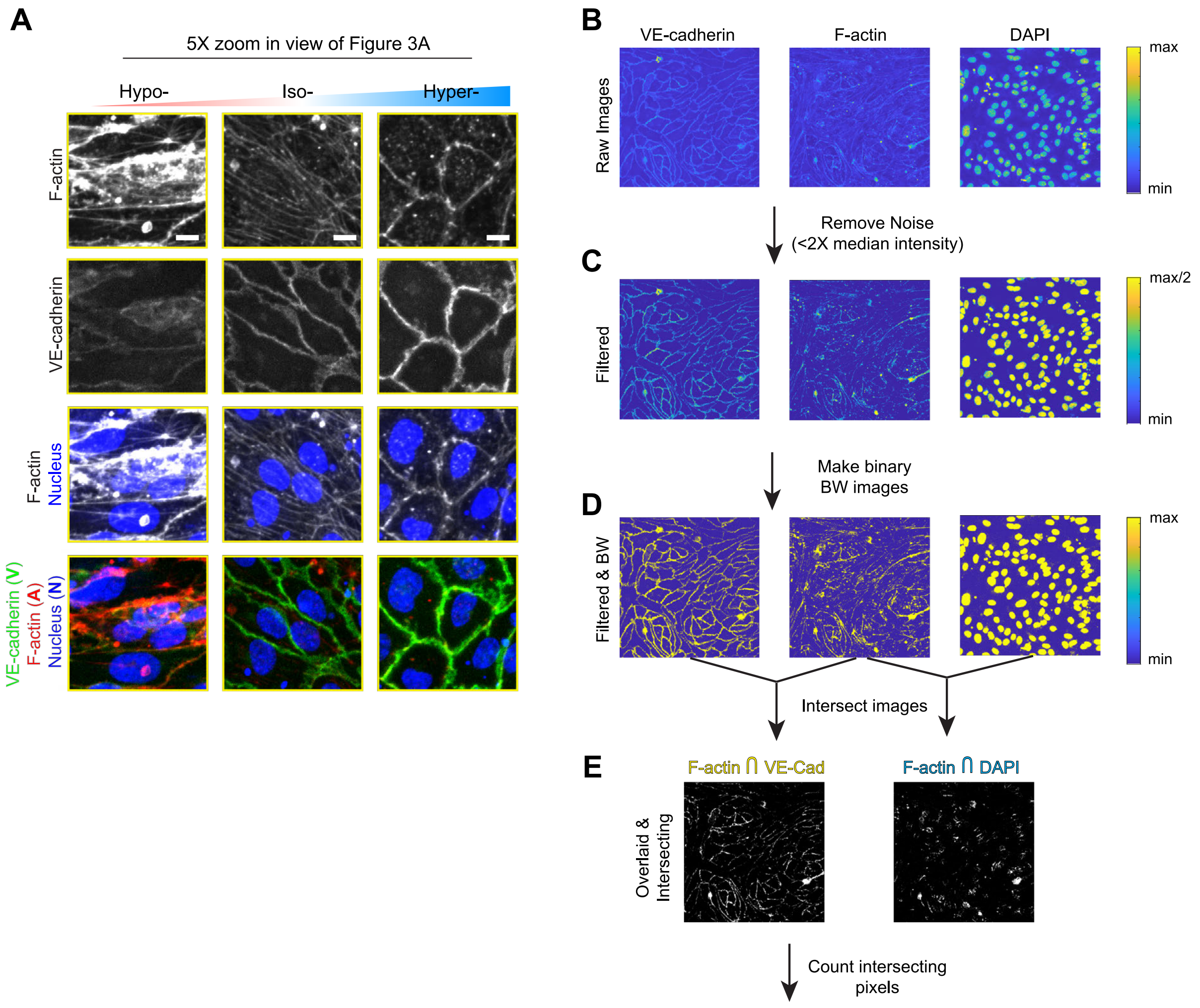

Figure S15. Illustration of the F-actin localization quantification.
A) Zoom-in view of Figure 3A (yellow boxes), which shows a representative immunostaining of F-actin and VE-Cadherin in HUVEC 2.5D monolayer 2 days after corresponding osmotic adjustment (hypo-, iso-, or hyper-osmotic condition at D2; see Figure 1B for detailed timelines). Scale bars, 10 µm. **B-E)** Successive steps of image analysis to obtain the fraction of F-actins localized in the cell-cell junction and cell body. 2.5D HUVEC monolayers in isoosmotic conditions are presented as an example. Raw images for each fluorescent channel are displayed (B). All pixels that are less than 2× median intensity of each channel is removed from the analysis (C). The filtered image is then transformed into binary black and white (BW) images (D). Pixels that are positive (white) in both channels (i.e., F-actin ∩ VE-cadherin or F-actin ∩ DAPI) are selected and plotted (E). The colocalization amount is then calculated by dividing the number of intersecting pixels by the total number of pixels in the filtered F-actin image shown in panel (C). See Experimental Section for additional details.

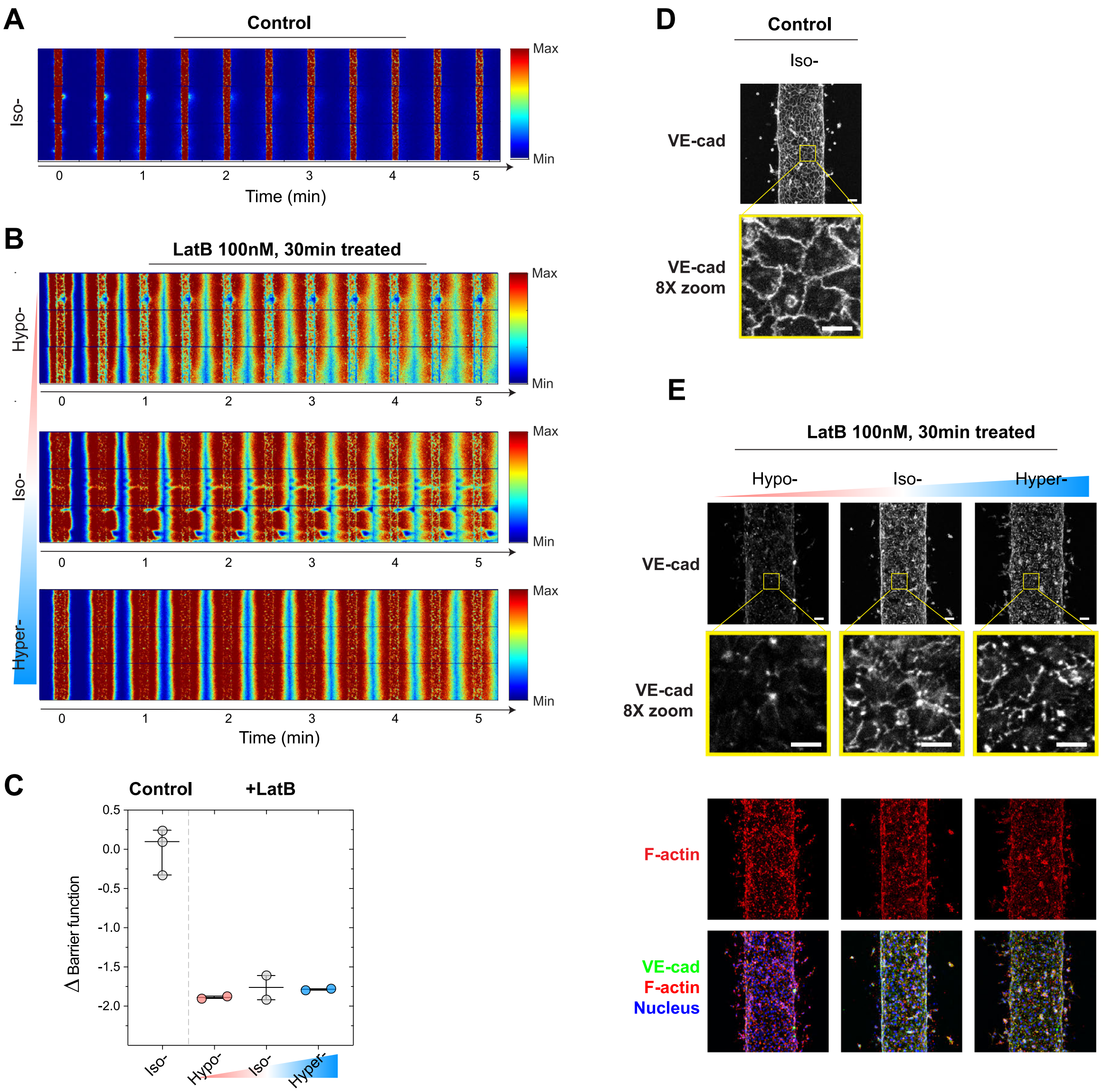

Figure S16. F-actins are required for maintaining the barrier function and adherens junction in all osmotic conditions.

A, B) Representative fluorescent images of 4 kDa FITC-Dextran leakage from iso- (A) and osmolarity-adapted (hypo-, iso-, or hyper-osmotic conditions) HUVEC 3D engineered microvessels after actin depolymerizing agent Latrunculin B (LatB) 100 nM treatment for 30 min (B). t=0 min images were taken immediately after the microvessel lumen was filled with 4kDa FITC-dextran. **C)** The barrier function of the conditions described in panels (A) and (B) (n=3, 2, 2, 2 microvessels from left to right). Box and whisker plots represent median value (horizontal bars), 25 to 75 percentiles (box edges), and minimum to maximum values (whiskers). **D)** Representative immunostaining of VE-cadherin in isoosmotic control HUVEC 3D engineered microvessels. **E)** Representative immunostaining of VE-cadherin and F-actin in osmolarity-adapted HUVEC 3D engineered microvessels after LatB 100 nM 30 min treatment. Note that the F-actins are no longer in filamentous form but rather displayed in blobs after LatB treatment. Cell nuclei were counterstained with DAPI. In panels (D and E), Scale bars, 50 µm. *Inset:* 8× zoom-in view of the marked yellow regions. Scale bars, 20 µm.

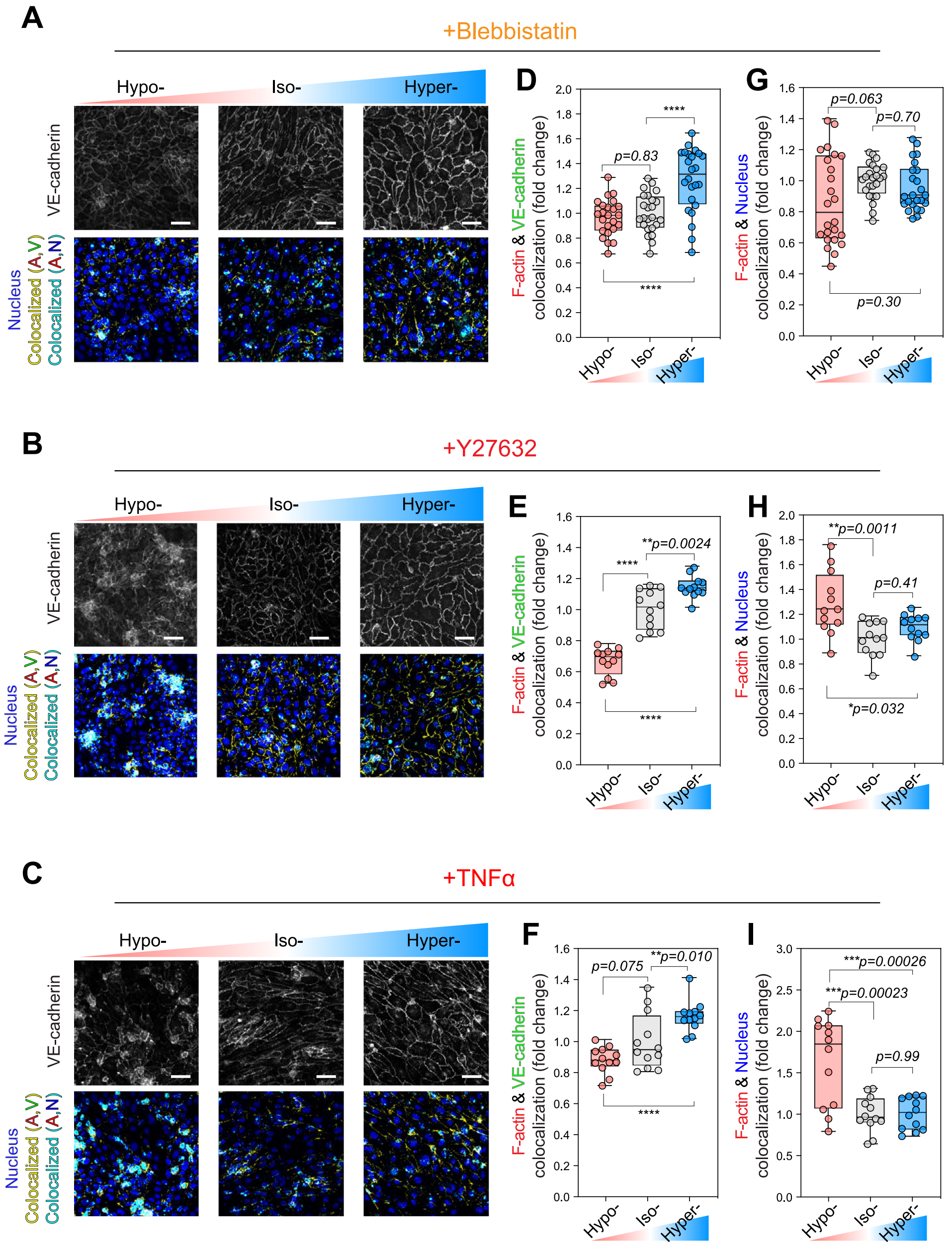

Figure S17. F-actin localization after ROCK inhibitor Y-27632, myosin II inhibitor Blebbistatin, and pro-inflammatory cytokine TNFα treatment.

A-C) Representative immunostaining of VE-cadherin (1st row) and co-localized pixels of F-actin & VE-cadherin (yellow; 2nd row), F-actin & nucleus (cyan; 2nd row) in osmolarity-adapted (hypo-, iso-, and hyper-osmotic conditions) human umbilical vein endothelial cell (HUVEC) 2.5D monolayers after Blebbistatin 10 µM 30 min (A), Y-27632 10 µM 30 min (B), and TNFα 5 ng/ml 24 h (C) treatment. Cell nuclei were counterstained with DAPI (blue). Scale bars, 50 µm. **D-I)** Boxplots displaying colocalization of F-actin & VE-cadherin (D to F) and F-actin & Nucleus (G to I) from the immunostaining images. n = 24, 12, and 12 cropped images from N = 2, 1, and 1 independent experiments for Blebbistatin (D and G), Y-27632 (E and H), and TNFα (F and I) treated samples, respectively. Box and whisker plots represent median value (horizontal bars), 25 to 75 percentiles (box edges), and minimum to maximum values (whiskers). P values were obtained using one-way ANOVA followed by Tukey's HSD post hoc test. ****P<0.0001.

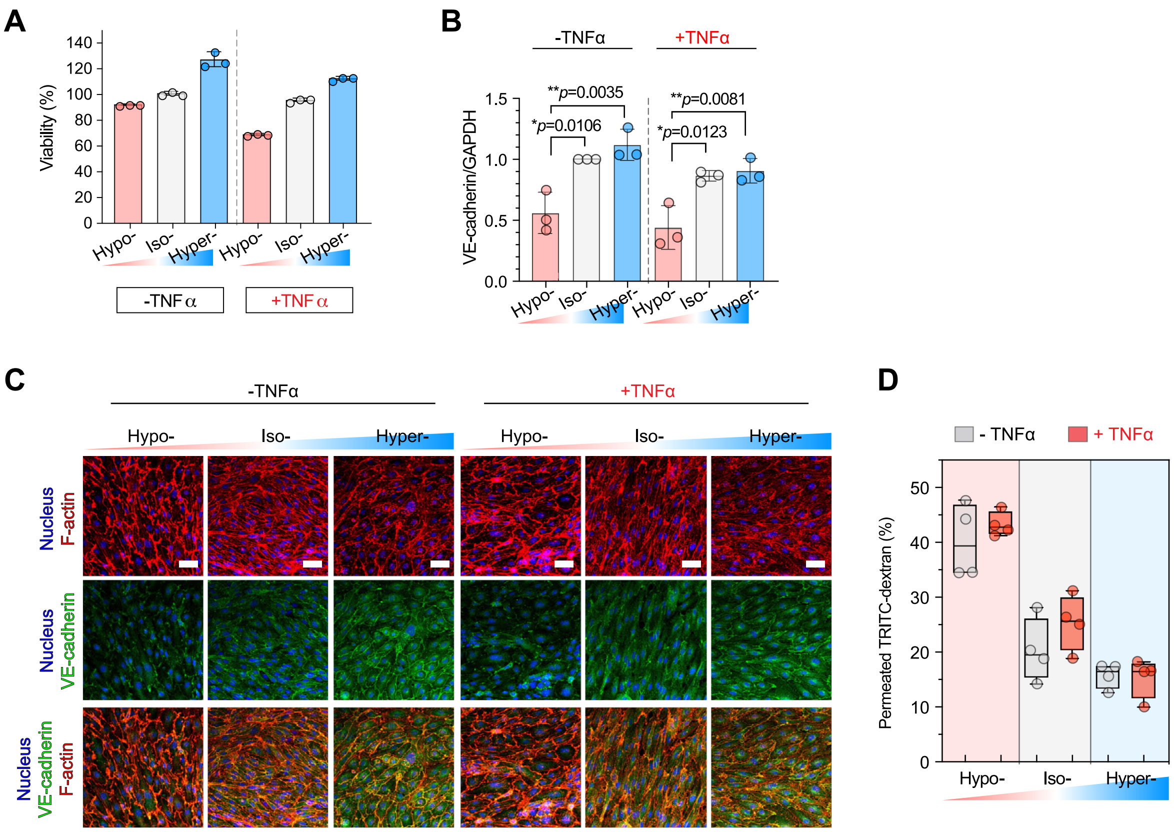

Figure S18. Effect of tumor necrosis factor-α (TNFα) treatment on endothelium viability, expression of VE-cadherin and Transwell permeability.

A) Viability of osmolarity-adapted (hypo-, iso-, and hyper-osmotic conditions) human umbilical vein endothelial cell (HUVEC) 2.5D monolayers with (+) or without (-) TNFα treatment. **B)** Quantitative normalization of VE-cadherin intensity level in osmolarity-adapted HUVEC 2.5D monolayers with or without TNFα 5 ng/ml 24 h treatment. GAPDH was used as a loading control. Graph in (A and B) represent mean ± S.D. n = 3 biological replicates. P values were obtained using one-way ANOVA followed by Tukey's HSD post hoc test. **C)** Representative immunostaining of VE-cadherin (green) and F-actin (red) in osmolarity-adapted HUVEC 2.5D monolayers, with or without TNFα 5 ng/ml 24 h treatment. Cell nuclei were counterstained with DAPI. Scale bars, 50 µm. **D)** The percentage of permeated TRITC-dextran through osmolarity-adapted HUVEC 2D monolayers with or without TNFα 5 ng/ml 24 h treatment. n = 4 wells for all conditions. Box and whisker plots in panel (D) represent median value (horizontal bars), 25 to 75 percentiles (box edges), and minimum to maximum values (whiskers).

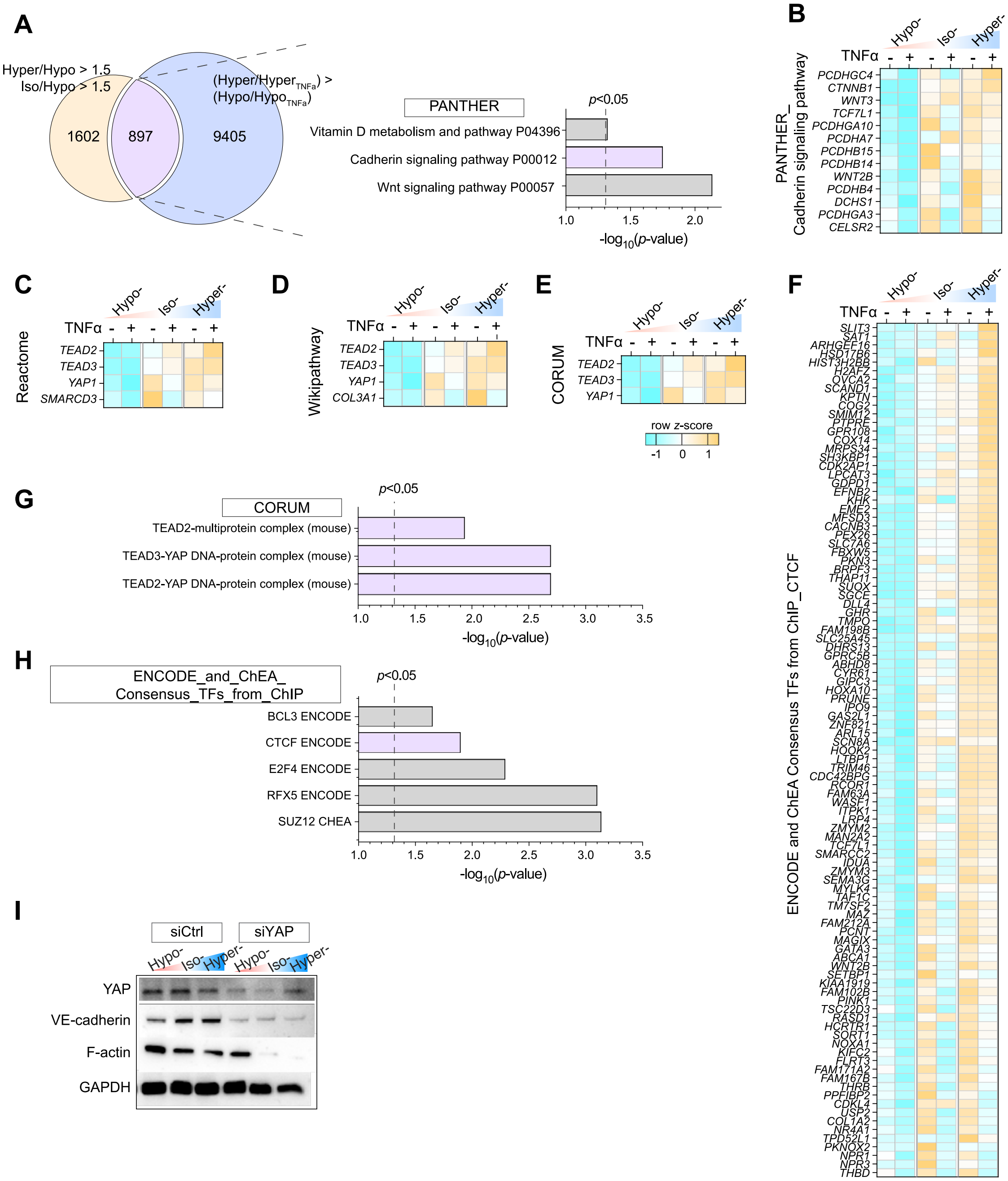

Figure S19. Yes-associated protein (YAP) and its related gene expression changes after osmolarity adaptation and TNF-a induced vascular inflammation.

A) Left*:* Number of genes that are upregulated by more than 1.5-folds in hyper- and iso- compared to hypo- (Hyper/Hypo >1.5 & Iso/Hypo >1.5) and that are highly maintained after TNFα treatment in hyper-, but not in hypo-osmotic conditions (Hyper/Hyper$_{TNF\alpha}$ > Hypo/Hypo$_{TNF\alpha}$). Same as in Figure 5A. Right*:* Gene Ontology (GO) analysis for the 897 intersected genes from PANTHER. **B-F)** Heatmap visualization of gene expression profiles from PANTHER (B), Reactome (C), Wikipathway (D), CORUM (E), and ENCODE and 'ChEA Concensus TFs from ChIP' (F) included in the 897 intersected genes. Heatmap analysis was visualized by the row z-score (n = 1). **G, H)** GO analysis for the 897 intersected genes from CORUM (G), and ENCODE and 'ChEA Concensus TFs from ChIP' (H). **(I)** Western blot displaying YAP, VE-cadherin and F-actin levels in YAP-depleted (siYAP) and control (siCtrl) 2.5D HUVEC monolayers after osmolarity adaptation (hypo-, iso-, and hyper-osmotic conditions). GAPDH was used as a loading control. In panels (A, G, and H)**,** the dashed vertical lines indicate significance at $p < 0.05$.

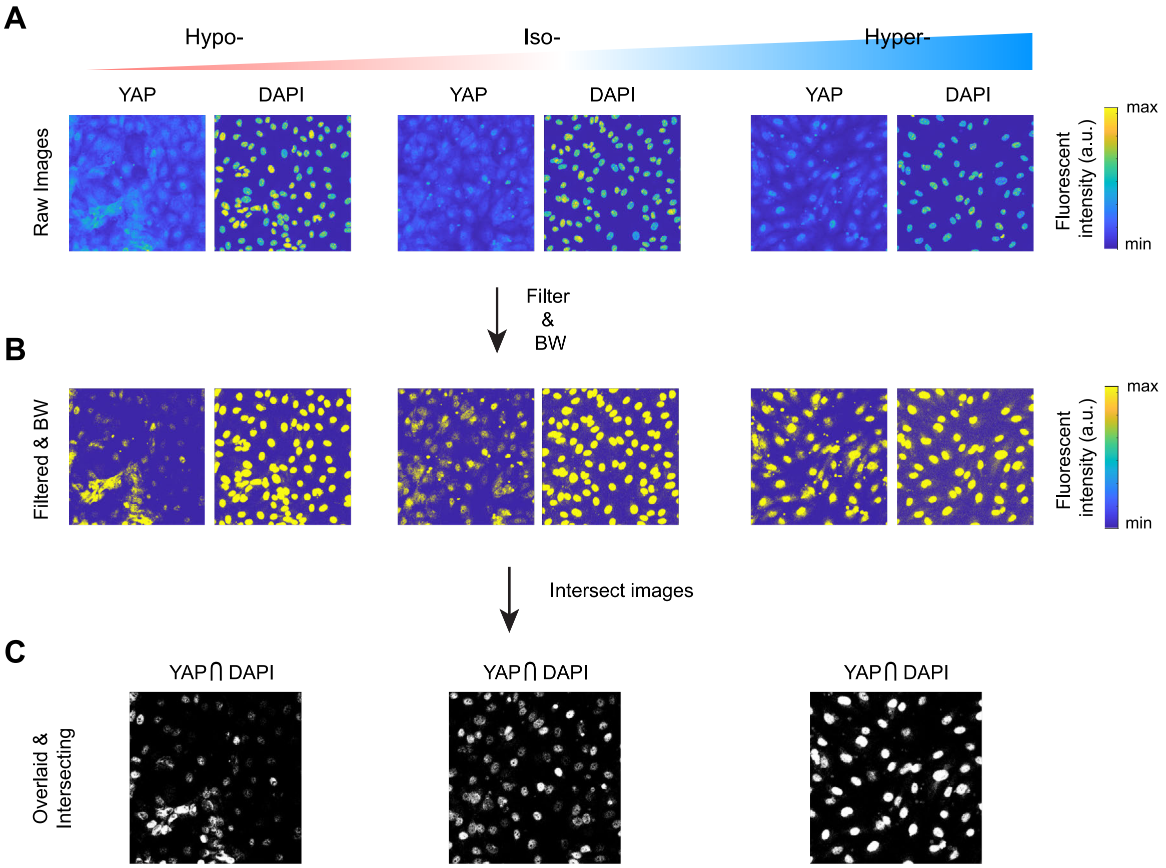

Figure S20. Illustration of the Yes-associated protein (YAP) nuclear localization quantification.
A-C) Successive steps of image analysis to obtain the YAP colocalization in osmolarity-adapted HUVEC monolayers. Raw images for each channel are displayed (A). All pixels that are less than 2× median intensity of each channel is removed from the analysis and the image is transformed into binary black and white (BW) images (B). Pixels that are positive (white) in both channels (i.e., YAP ∩ DAPI) are selected and plotted (C). The colocalization amount shown in Figure 5E was calculated by dividing the number of intersecting pixels by the total number of pixels in raw YAP images rather than the number of pixels in the filtered images, due to the low signal-to-noise ratio in YAP immunostaining images. See Experimental Section for additional details.
